# A novel renal perivascular mesenchymal cell subset gives rise to fibroblasts distinct from classic myofibroblasts

**DOI:** 10.1038/s41598-022-09331-5

**Published:** 2022-03-30

**Authors:** Shun Minatoguchi, Shoji Saito, Kazuhiro Furuhashi, Yuriko Sawa, Masaki Okazaki, Yuko Shimamura, Ahmad Baseer Kaihan, Yusaku Hashimoto, Yoshinari Yasuda, Akitoshi Hara, Yasuyuki Mizutani, Ryota Ando, Noritoshi Kato, Takuji Ishimoto, Naotake Tsuboi, Nobutoshi Esaki, Makoto Matsuyama, Yukihiro Shiraki, Hiroki Kobayashi, Naoya Asai, Atsushi Enomoto, Shoichi Maruyama

**Affiliations:** 1 Department of Nephrology, Nagoya University Graduate School of Medicine, 65 Tsurumai-Cho, Showa-Ku, Nagoya, Aichi 466-8550 Japan; 2 Department of Pathology, Nagoya University Graduate School of Medicine, Nagoya, Aichi Japan; 3 Department of Nephrology, Graduate School of Medicine, Fujita Health University, Toyoake, Aichi Japan; 4 Division of Molecular Genetics, Shigei Medical Research Institute, Okayama, Japan; 5 Department of Molecular Pathology, Graduate School of Medicine, Fujita Health University, Toyoake, Aichi Japan

**Keywords:** Kidney, Kidney diseases, Cell biology

## Abstract

Perivascular mesenchymal cells (PMCs), which include pericytes, give rise to myofibroblasts that contribute to chronic kidney disease progression. Several PMC markers have been identified; however, PMC heterogeneity and functions are not fully understood. Here, we describe a novel subset of renal PMCs that express Meflin, a glycosylphosphatidylinositol-anchored protein that was recently identified as a marker of fibroblasts essential for cardiac tissue repair. Tracing the lineage of Meflin^+^ PMCs, which are found in perivascular and periglomerular areas and exhibit renin-producing potential, showed that they detach from the vasculature and proliferate under disease conditions. Although the contribution of Meflin^+^ PMCs to conventional α-SMA^+^ myofibroblasts is low, they give rise to fibroblasts with heterogeneous α-SMA expression patterns. Genetic ablation of Meflin^+^ PMCs in a renal fibrosis mouse model revealed their essential role in collagen production. Consistent with this, human biopsy samples showed that progressive renal diseases exhibit high Meflin expression. Furthermore, Meflin overexpression in kidney fibroblasts promoted bone morphogenetic protein 7 signals and suppressed myofibroblastic differentiation, implicating the roles of Meflin in suppressing tissue fibrosis. These findings demonstrate that Meflin marks a PMC subset that is functionally distinct from classic pericytes and myofibroblasts, highlighting the importance of elucidating PMC heterogeneity.

## Introduction

Renal fibrosis is a mechanism underlying chronic kidney disease (CKD), whose prevalence is increasing worldwide^1–3^. In renal fibrosis, myofibroblasts proliferate and produce extracellular matrix, inducing a vicious cycle of CKD progression. Previous studies have shown that myofibroblasts, characterized by α-smooth muscle actin (α-SMA) expression, are derived from various types of cells, such as resident fibroblasts, circulating fibrocytes, and epithelial and endothelial cells^4–8^. Nevertheless, the exact origin of myofibroblasts is still under discussion, and the possibility that myofibroblasts are derived from other cells, such as perivascular mesenchymal cells (PMCs), has been suggested^9–13^.

PMCs are associated with the vasculature and are present in various renal areas, including the glomerular mesangial and peri-glomerular areas as well as the interstitium around renal tubules^14–16^. PMCs consist of heterogeneous cell populations including classic pericytes, perivascular fibroblasts, adventitial cells, and vascular smooth muscle cells^11,12,16^. Previous studies have shown that PMCs play multiple roles in kidney tissue homeostasis. For example, glomerular mesangial cells function as structural support for the glomerular capillary loop and regulate glomerular blood flow^17,18^. Renin-producing cells at the glomerular vascular pole are another subset of PMCs^19,20^. Notably, PMCs play a central role in the etiology of renal fibrosis^12^; a small fraction of renal PMCs (0.01% of all kidney cells) expresses Gli1, which is essential for the Sonic Hedgehog signaling pathway, and gives rise to α-SMA^+^ myofibroblasts that contribute to renal fibrosis^21,22^.

Interestingly, recent single-cell transcriptomic analyses of multiple organs have revealed that fibroblasts and pericytes are heterogeneous, suggesting that the kidneys also contains various types of PMCs^23,24^. PMC markers identified so far include platelet-derived growth factor receptor B (PDGFRβ), chondroitin sulfate proteoglycan 4 (CSPG4)^25^, melanoma cell adhesion molecule (MCAM/CD146)^26^, and α-SMA^27^. PDGFRα, desmin, 5'-nucleotidase ecto, and regulator of G protein signaling 5 (RGS5) are also employed as pericyte markers^11^. However, these markers are expressed across various types of PMCs and classic pericytes, and most are not specific for PMCs. In addition, the molecular functions of these PMC markers in renal homeostasis, fibrosis, and tissue repair are not completely understood. Therefore, more studies are needed to fully elucidate the heterogeneity of PMCs and their functional significance.

We previously identified Meflin, a glycosylphosphatidylinositol (GPI)-anchored membrane protein encoded by the immunoglobulin superfamily containing a leucine-rich repeat (*Islr*) gene, as a specific marker of undifferentiated mesenchymal stromal/stem cells (MSCs)^28^. Consistent with the emerging notion that MSCs share a number of characteristics with pericytes or perivascular cells^29^, Meflin^+^ cells are found in the perivascular areas of multiple organs including the bone marrow, pancreas, heart, skeletal muscles, and skin, but not in these organs’ epithelial, endothelial, smooth muscle, immune, or blood cells^28^. Interestingly, previous studies revealed that Meflin^+^ cells proliferate during chronic cardiac diseases, where Meflin functions in tissue repair after acute myocardial infarction and has an anti-fibrotic role by augmenting the signaling of bone morphogenetic protein (BMP) 7; BMP7 is known to counteract the pro-fibrotic transforming growth factor β (TGF-β) pathway^30,31^. Meflin^+^ cells also proliferate to constitute a subset of cancer-associated fibroblasts (CAFs) in the stroma of pancreatic and lung cancer, where Meflin suppresses cancer progression by inhibiting the remodeling of collagen architecture in the stroma and modulating tumor vessel architecture^32–34^, and colorectal cancer, where Meflin promote BMP signaling^35^. Thus, Meflin^+^ cells can suppress the progression of chronic diseases, which is different from those of conventional α-SMA^+^ myofibroblasts or CAFs that promote those diseases. Other lines of evidence indicate that Meflin also suppresses pulmonary fibrosis and is involved in skeletal muscle and intestinal regeneration^36–39^. However, the functions of Meflin protein and Meflin^+^ cells in the kidneys remain unknown.

This study aimed to investigate the role of Meflin protein and Meflin^+^ cells in the kidneys. First, we localized and characterized Meflin^+^ cells in the normal kidney using a Meflin reporter mice line and publicly available gene expression data. We also conducted in situ hybridization (ISH) analysis for Meflin (*Islr*) and other PMC makers. To investigate the function of Meflin^+^ cells, the effects of genetic ablation of Meflin^+^ cells on renal homeostasis were examined. Next, we investigated the role Meflin^+^ cells in renal disease conditions by studying a unilateral ureteral obstruction (UUO) mouse model, a model for renal fibrosis. Further, lineage tracing experiments using Meflin reporter mice line was conducted to unveil proliferation of Meflin^+^ cells during renal fibrosis. In addition, human renal biopsy samples from patients with minimal change nephrotic syndrome (MCNS), diabetic kidney disease (DKD), and IgA nephropathy (IgAN) were analyzed. Finally, we examined the molecular function of Meflin using cultured kidney fibroblasts.

## Methods

### Study approval

All animal protocols were approved by the Animal Care and Use Committee of Nagoya University Graduate School of Medicine (approval number: 20351). All the in vivo experiments were performed in compliance with Nagoya University’s Animal Facility regulations. The study was conducted in accordance with the Declaration of Helsinki for Human Research and approved by the Ethics Committee of Nagoya University Graduate School of Medicine. All patients provided written informed consent for participation in the Nagoya Kidney Disease Registry (N-KDR), which was approved by the Ethics Committee of Nagoya University Graduate School of Medicine (approval number: 2010-1135). The study is reported in accordance with ARRIVE guidelines.

### Animals

Meflin-Zsgreen-t2a-diphtheria toxin receptor (DTR)-t2a-Cre (ZDC) and Meflin-CreER^T2^ knock-in mice were generated as previously described^30,32^. Meflin-ZDC and Meflin-CreER^T2^ mice were crossed with Rosa26-LSL (*Lox*P-stop-*Lox*P)-tdTomato mice (JAX stock number 007909) to generate Meflin-CreER^T2^; Rosa26-LSL-tdTomato mice and enable lineage tracing. Knock-in mice were mated with C57BL/6 mice to generate mice with a C57BL/6 genetic background. Genomic DNA extracted from mouse tails were used for PCR genotyping using the following primers: WT forward, 5′-ACACACGACCTTGGCAAGTCCCAGC-3′; WT reverse, 5′-GTCTGCAATCTGGAAGCCATACTTCTCC-3′; Meflin-ZDC forward, 5′-TAGGTGGTATTGGATTCTGGCTGGG-3′; Meflin-ZDC reverse, 5′-TTGAAGTAGTCGACGATGTCCTGG-3′; Meflin-CreER^T2^ forward, 5′-ACACACGACCTTGGCAAGTCCCAGC-3′; and Meflin-CreER^T2^ reverse, 5′-CGATCCCTGAACATGTCCATCAGG-3′. PCR product sizes from WT, Meflin-ZDC, and Meflin-CreER^T2^ alleles were 291, 385, and 359 bp, respectively. Meflin KO mice were also generated as previously described^28^ and PCR genotyping was performed using the following primers: PCR1 forward, 5′-GCTGCATTTGAGCTGAGCCTCTGG-3′; PCR1 reverse, 5′-AACCCCTTCCTCCTACATAGTTGG-3′; PCR2 forward, 5′-TGAGGTTAGCCTGGGGACTTCAC-3′; and PCR2 reverse, 5′-GGCTAGAACTCTCAAAGTAGGTCAGG-3′.

All mice were maintained in standard clean cages. Water and diet for mice were available ad libitum throughout the experimental period. Isoflurane (2–3%) was used for anesthesia during surgery and kidney extraction. After kidney extraction, the mice were euthanized by cervical dislocation.

### Genetic tracing of Meflin lineage cells

To analyze PMCs expressing Meflin in Meflin-CreER^T2^; LSL-tdTomato mice, 6–10-week-old mice were i.p. administered 0.1 mg/g tamoxifen (TAM, T5648; Sigma-Aldrich, St. Louis, MO) every other day for a total of 10 times. Two days after the last TAM injection, the kidneys were harvested and fixed. For lineage tracing analysis, 6–10-week-old Meflin-CreER^T2^; LSL-tdTomato mice were i.p. administered 0.1 mg/g TAM every other day for a total of 10 times, followed by a 2-week washout period before UUO surgery. Seven days after UUO surgery, mice were sacrificed and their kidneys were harvested. For the experiment examining the relationship between tdTomato^+^ cells and vasculatures, 10 mg TAM per mouse was administered orally every other day for a total of 3 times. After a two-week washout period, mice were subjected to UUO surgery and sacrificed on days 1, 3, and 7 after surgery. To examine the involvement of Meflin^+^ cells in renin production via lineage tracing, mice were orally administered 10 mg TAM per mouse every other day for a total of 3 times, followed by a 2-week washout period, and then fed a low-salt diet (0.02% NaCl; CLEA Japan, Inc., Tokyo, Japan) and captopril (0.5 g/L in drinking water; Sigma-Aldrich)^40,41^. Mice were sacrificed on day 7 after the start of the low-salt diet and their kidneys were harvested.

### Vasculature staining and tissue clearing

For the vasculature staining, 40 µL anti-CD31 (500 µg/mL; 102516; BioLegend, San Diego, CA), 40 µL anti-Isolectin B4 (1000 µg/mL; I32450; Invitrogen, Carlsbad, CA), and 220 µL phosphate-buffered saline (PBS) were intravenously injected 1 h before kidney harvesting procedure to visualize vascular endothelial cells^42^, followed by fixation with a paraformaldehyde (PFA) buffer. For optical tissue clearing, we employed the SeeDB2 method^43^. Imaging was performed using an A1RMP confocal microscope (Nikon, Tokyo, Japan) and processed with Imaris software (v8.4.2; Bitplane, Zurich, Switzerland).

### ISH

ISH was performed as previously described^32^; briefly, 10% formalin-fixed, paraffin-embedded mouse kidney tissue samples or Masked-form A18 (NTS00102; Japan Tanner Corporation, Osaka, Japan)-fixed paraffin-embedded human kidney biopsy samples were stained using RNAscope technology (RNAscope 2.5 HD Detection Kit, RNAscope 2.5 Duplex Detection Kit and RNAscope Multiplex Fluorescent Reagent Kit v2; Advanced Cell Diagnostics, Newark, CA) following the manufacturer’s instructions. The following RNAscope probes were used: human Meflin *(ISLR*; catalog no. 455481; NM_005545.3, target region 275–1322); mouse Meflin *(Islr*; catalog no. 450041; NM_012043.4, target region 763–1690); mouse Meflin *(Islr*; catalog no. 453321-C2; NM_012043.4, target region 277–2225); mouse *Acta2* (catalog no. 319531-C2; NM_007392.3, target region 41–1749); mouse *Gli1* (catalog no. 311001; NM_010296.2, target region 25–1205); mouse *Cspg4* (catalog no. 404131-C2; NM_139001.2, target region 1431–2308); mouse *Pdgfrb* (catalog no. 448421; NM_001146268.1, target region 3622–4657); mouse Pdgfra (catalog no. 480661-C2; NM_011058.2, target region 223–161); mouse Epo (catalog no. 315501-C2; NM_007942.2, target region 39–685); and mouse Ren1 (catalog no. 433461-C2; NM_031192.3, target region 5–1410) ; mouse Col1a1 (catalog no. 319371; NM_007742.3, target region 1686–4669); tdTomato (catalog no.317041-C2; target region 7–1382) and mouse Col3a1 (catalog no. 455771; NM_009930.2, target region 873–1711).

For the ISH in Fig. S3b, which is also described previously^32^, mouse kidney tissues were dissected, treated with Tissue Fixative (Genostaff, Tokyo, Japan), embedded in paraffin, and sectioned at 4–8 μm. Tissue sections were then dewaxed with xylene and rehydrated with an ethanol series and PBS wash. The sections were fixed with 4% PFA for 15 min, washed with PBS, treated with Proteinase K (8–10 μg/mL) in PBS for 30 min at 37 °C, washed with PBS, re-fixed with 4% PFA, washed with PBS, and then incubated in 0.2 N HCl for 10 min. After washing with PBS, the sections were acetylated by incubation in 0.1 M triethanolamine-HCl (pH 8.0, 0.25% acetic anhydride) for 10 min, washed again with PBS, dehydrated using a series of ethanol rinses, and hybridized with probes (300 ng/mL) in Probe Diluent-1 (Genostaff) for 16 h at 60 °C. After hybridization, the sections were washed in 5× HybriWash (Genostaff) for 20 min at 60 °C and in 50% formamide and 2× HybriWash for 20 min at 60 °C, followed by treatment with RNase [50 μg/mL RNaseA, 10 mM Tris–HCl (pH 8.0), 1 M NaCl, and 1 mM EDTA) for 30 min at 37 °C. Samples were then washed twice each for 20 min at 60 °C with 2× and 0.2× HybriWash and then once with TBST (0.1% Tween 20 in TBS). After treatment with 1× G-Block (Genostaff) for 15 min at room temperature, the sections were incubated with anti-digoxigenin-alkaline phosphatase (DIG AP) conjugate (Roche, Basel, Switzerland) diluted 1:2000 with 0.02X G-Block in TBST for 1 h at room temperature, washed twice with TBST, and incubated in 100 mM NaCl, 50 mM MgCl2, 0.1% Tween 20, and 100 mM Tris–HCl (pH 9.5). Color reactions were performed with nitroblue tetrazolium and 5′-bromo-4-chloro-3-indolyl phosphate (NBT/BCIP) solution (Sigma-Aldrich) overnight. The sections were counterstained with Kernechtrot stain solution (Muto pure chemicals, Tokyo, Japan) and mounted with CC/Mount (Diagnostic Biosystems, Pleasanton, CA).

### Flow cytometry

For flow cytometry, Meflin-CreER^T2^; LSL-tdTomato mice were sacrificed after i.p. administration of 0.1 mg/g TAM performed every other day for a total of 10 times, and then their kidneys were harvested. The kidneys were minced and digested with a digestion buffer (1 mg/mL type 1 collagenase, 0.1 mg/mL DNAse in Hank’s balanced salt solution) to obtain single cell suspensions. Following treatment with a red blood cell (RBC) lysis buffer (BioLegend), the cell suspensions were plated onto 96-well plates and incubated with the following antibodies for 30 min: anti-CD45-Pacific Blue (cat. no. 103126, clone 30-F11; BioLegend), anti-CD31-PE/Cy7 (cat. no. 102418, clone 390; BioLegend), anti-CD146-FITC (cat. no. 134706, clone ME-9F1; BioLegend), anti-CD140a-Alexa647 (cat. no. 562777, clone APA5; BD Biosciences, Franklin Lakes, NJ), anti-CD140b-Biotin (cat. no. 136010, clone APB5; BioLegend), and anti-Sca1-Brilliant Violet 605 (cat. no. 108134, clone D7; BioLegend). Flow cytometry was performed using a FACSAria Fusion cytometer (BD Biosciences), followed by data analysis using FlowJo software (Tree Star Inc., Ashland, OR).

### Analysis of publicly accessible omic datasets

Gene expression data of cell-type specific genes (accession number: GSE52004)^44^, adult JG complex cells and renal cortex (GSE42713)^45^, and PDGFRα^+^ fibroblasts in mouse disease models (GSE121190)^46^ were all obtained from the Gene Expression Omnibus (GEO). Expression data were analyzed with GEO2R (RRID:SCR_016569), an interactive web tool in GEO, and visualized using the ggplot2 package in R software (v4.0.1)^47^. Multi-omics data of renal disease mouse models were processed using the Mouse Kidney FibrOmics browser (http://hbcreports.med.harvard.edu/fmm/)^48^. Single-cell RNA-seq dataset of adult mouse kidney (GSE129798)^49^ and human allograft kidney tissues undergoing clinical rejection (GSE109564)^50^ were obtained from the GEO. Data processing was carried out using the Seurat^51^, ggplot2^52^, and dplyr^53^ packages in R. After removing unwanted cells as described in the cited articles, a global-scaling normalization method was used to normalize the feature expression measurements for each cell by the total expression, which was multiplied by a scale factor (10,000 by default), followed by log-transformation of the result. Next, we performed linear dimension reduction using principal component analysis. The useful principle components were determined by the visualization tool ElbowPlot, and cells were clustered using a graph-based approach using FindClusters at appropriate resolutions. To visualize and explore these datasets, UMAP was applied to place similar cells together in two-dimensional spaces. Stromal cells were extracted based on *Pdgfrb* expression, and then re-clustered and visualized with UMAP plotting. We also visualized gene expression using Dotplot and Featureplot where necessary.

### qPCR

Total RNA was extracted using TRIzol reagent (Invitrogen), followed by cDNA generation from purified RNA using the PrimeScript RT Reagent Kit (Perfect real time; Takara Bio, Shiga, Japan). qPCR was performed with the PowerUp SYBR Green Master Mix or TaqMan Gene Expression Master Mix on the StepOnePlus Real Time PCR system (all Applied Biosystems; Foster City, CA). The primers were purchased from Sigma-Aldrich *(Islr, Acta2, Col1a1, Ren1,* and *Akr1b7*) and IDT Inc. (PrimeTime qPCR Primers for *Ngal* and *HsHBEGF*). Primer sequences were as follows: mouse *Islr* sense, 5′-GCCAAAGCCTGCTTGTTCTC-3′ and antisense, 5′-TCACCAATTCCAGACCTCGTG-3′; mouse *Acta2* sense, 5′-CTCTTCCAGCCATCTTTCATTG-3′ and antisense, 5′-GTTGTTAGCATAGAGATCCTTCCT-3′; mouse *Col1a1* sense, 5′-ATGGATTCCCGTTCGAGTACG-3′ and antisense, 5′-TCAGCTGGATAGCGACATCG-3′; mouse *Ren1* sense, 5′-GTCCTCACCAACTACCTGAATACC-3′ and antisense, 5′-CACCCAGAGGTTGGCTGAAC-3′; mouse *Akr1b7* sense, 5′-GCCACCCTTATCTCTCACCCAG-3′, and antisense, 5′-TCTCCATTACTACGGGGTCTTC-3′; mouse *Ngal* (*Lcn2*) sense, 5′-CTACAATGTCACCTCCATCCTG-3′, and antisense, 5′-CCTGTGCATATTTCCCAGAGT-3′; and human *HBEGF* sense, 5′-TACCTATGACCACACAACCATC-3′, and antisense, 5′-GCCCAACTTCACTTTCTCTTCA-3′. TaqMan probes and primers for rat Meflin (*Islr*; Rn01766566_g1), rat α-SMA (*Acta2*; Rn1759928_g1), rat vimentin (*Vim*; Rn00667825_m1), rat DNA-binding protein inhibitor ID-1 (*Id1*; Rn00562985_s1), rat Plasminogen activator inhibitor-1 PAI-1 (*Serpine1*; Rn01481341_m1) , rat Collagen type I alpha 1 chain Col1a1 (*Col1a1*; Rn01463848_m1), and rat glyceraldehyde-3-phosphate dehydrogenase (*Gapdh*; Rn01775763_g1) were purchased from Thermo Fisher Scientific (Waltham, MA). The expression data were analyzed using the 2^−ΔΔCt^ method and normalized to *Gapdh* expression.

### Genetic ablation of Meflin^+^ cells

For ablating Meflin^+^ PMCs, Meflin-ZDC mice were i.p. injected with diphtheria toxin (DTx, 3 ng/g) once. As control, we injected WT(C57BL6/J) and Meflin-ZDC mice with the same dose of DTx and PBS, respectively. Three days after DTx administration, mice were sacrificed. Meflin-ZDC mice were also i.p. injected with DTx (1.5 ng/g) on days 1, 3, and 5 after UUO surgery; PBS-injected Meflin-ZDC mice were used as controls and mice were sacrificed 10 days after surgery. Finally, Meflin-ZDC mice were injected with DTx (2 ng/g) and sacrificed on days 3 and 6. Owing to the lethal effect of the genetic ablation of Meflin^+^ PMCs, DTx dosage for long-term experiments were reduced to 1.5 and 2 ng/g, respectively. In all experiments, mice were fed with Dietgel 76A and Dietgel Boost (ClearH2O, Portland, ME) to reduce intestinal damage associated with the ablation of intestinal Meflin^+^ cells.

### Mouse models of kidney fibrosis

UUO models were established as previously described^54^. Briefly, after mice were sedated using isoflurane, an incision was made in the left side of the back, and complete ureteral obstruction was performed by double-ligating the left ureter using 5-0 silk. For the adenine-induced kidney injury models, mice were fed a 0.2% adenine-containing diet (Research Diets, Inc., New Brunswick, NJ) for 14 days. Mice were sacrificed at the indicated time points after surgery or diet, and their kidneys were harvested for subsequent examination.

### IHC, IF, and histology

For IHC of mouse tissues, formalin-fixed, paraffin-embedded tissue sections were deparaffinized and stained using conventional procedures with diaminobenzidine (DAB) as a chromogen. For human kidney biopsy samples, masked-form A18-fixed paraffin-embedded tissues were deparaffinized and stained using the Ventana Discovery Ultra system (Roche Diagnostics, Indianapolis, IN). Antibodies used were rabbit polyclonal anti α-SMA antibody (1:100; ab5694; Abcam, Cambridge, UK) and anti-human Meflin antibody (1:300; generated in the present study as described below). Images were acquired using an optical microscope (BX53; Olympus, Tokyo, Japan) equipped with a CCD digital camera (DP22; Olympus).

For IF, PFA-embedded tissue sections were deparaffinized and incubated with the following primary antibodies for 30 min to 2 h at room temperature: rabbit monoclonal anti-PDGFRβ (1:100; ab32570; Abcam), rabbit polyclonal anti-α-SMA (1:100; ab5694; Abcam), and rabbit monoclonal anti-Renin (1:50; ab212197, Abcam). The samples were then washed with PBS, incubated with Alexa Fluor 488- (Invitrogen) or FITC- (Jackson ImmunoResearch, West Grove, PA) conjugated secondary antibodies for 30 min at room temperature, and finally mounted using VECTASHIELD Mounting Medium with DAPI (Vector Laboratories, Burlingame, CA). Samples were imaged using an inverse immunofluorescence microscope (Axio Imager M2; Carl Zeiss, Oberkochen, Germany).

### Development of anti-human Meflin antibody

An anti-human Meflin monoclonal antibody was developed against recombinant full-length human Meflin using a proprietary method (Japanese Patent JP4098796B2). Briefly, the antigen emulsion, which included recombinant human Meflin^32^, was injected intramuscularly at the tail base of female WKY/NCrj rats. Rats were sacrificed 17 days after injection, and lymphocytes isolated from the iliac lymph nodes were fused with SP2/O-Ag14 mouse myeloma cells and cultured in 96-well plates. Eight days after cell fusion, culture supernatants were collected and screened for positive clones by solid-phase enzyme-linked immunosorbent assay. A clone was selected based on its specificity and sensitivity for human Meflin, which was assessed by IHC assays^32^, and used in the present study. We validated the specificity of the anti-Meflin antibody using Western blot analysis on lysates prepared from HEK293 cells transfected either with control vector or Meflin-expressing vector, which revealed that the Meflin antibody specifically detected exogenously-expressed Meflin in HEK293 cells (Fig. S1).

### Analysis of Meflin expression in human renal biopsy samples

To assess Meflin expression in human chronic kidney diseases, we reviewed consecutive patients who underwent diagnostic renal biopsy in 2019 from multiple centers. Inclusion criteria were: > 18 years old; diagnosed with either MCNS, IgAN, or DKD; non-dialysis patients; cortex rate > 50%; and glomerular counts > 10. A total of 41 patients were included.

To evaluate the relationship between Meflin expression and renal prognosis, we reviewed consecutive patients diagnosed with IgAN by renal biopsy from 2002 to 2012 at Nagoya University Hospital. After excluding patients with insufficient renal biopsy specimens, < 1 year of follow-up, and IgA vasculitis diagnosis, 99 patients were identified as eligible for the study.

IHC semi-quantification of Meflin levels in the biopsy samples was performed based on a scoring system employing overall Meflin score (0, Meflin^+^ PMCs detected only in glomerular vascular poles or interstitial area; 1, detected in < three regions; 2, detected in ≥ three regions; 3, diffuse proliferation of Meflin^+^ PMCs), Meflin interstitial score (0, no Meflin^+^ PMCs or non-specific Meflin signal in the interstitium; 1, Meflin^+^ PMCs detected in one region; 2, detected in two regions; 3, detected in ≥ three regions), and Meflin glomerular score (0, no Meflin^+^ PMCs or non-specific Meflin signal in the periglomerular areas; 1, Meflin^+^ PMCs detected in one region; 2, detected in two regions; 3, detected in ≥ three regions).

### Cell culture

Mouse kidney fibroblasts were isolated and cultured as previously described with some modifications^46^. Briefly, the mouse kidneys were harvested 3 days after UUO surgery, and the cortex diced and digested in 0.05% trypsin/EDTA for 10 min at 37 °C. The kidney pieces were placed in gelatin-coated dishes and incubated in DMEM/F12 with Glutamax (Gibco; Thermo Fisher Scientific) supplemented with 20% FBS, 1× Insulin-Transferrin-Selenium-Ethanolamine (ITS-X; Gibco), 5 µM Y-27632 (Enzo Life Sciences, Farmingdale, NY), and 1% penicillin/streptomycin for 3 days, after which the kidney pieces were gently removed and the medium was changed. The culture medium was renewed twice weekly. To examine the effect of TGF-β stimulation on gene expression, fibroblasts were cultured in 6-well plates to confluency, followed by serum starvation for 12 h before adding 10 ng/mL human recombinant TGF-β (R&D systems, Minneapolis, MN). Similarly, we stimulated the rat kidney fibroblasts cell line (NRK-49F; American Type Culture Collection, Manassas, VA) with TGF-β or BMP7 (R&D systems) but serum starvation was performed for 24 h before adding stimulation. Cells were harvested after 24 h and total RNA was extracted using TRIzol reagent.

### Plasmids

Cloning of mouse Meflin (mMeflin) cDNA was performed as previously described^28^. mMeflin cDNA was subcloned into pRetroQ (Clontech, Mountain View, CA) vectors without fusion with any tag sequences (pRetroQ-mMeflin).

### Expression of Meflin by a retroviral expression system

GP2-293 packaging cells (Clontech) seeded in collagen type I-coated 100-mm cell culture dishes were transfected with the pVSV-G (vesicular stomatitis virus G protein) vector and either control (pRetroQ-AcGFP) or pRetroQ-mMeflin vector using Lipofectamine 2000 reagent (Thermo Fisher Scientific). The medium was replaced after 24 h, and virus-containing supernatants were harvested 48 h post-transfection and used for infecting NRK-49F cells.

### Quantification of images and data visualization

Cell counting, measurement of vascular wall thickness and lumen size, and quantification of DAB staining were performed using Fiji software^55^. For morphological analysis, we used skeleton analysis^56^. Briefly, tdTomato^+^ cells observed in five fields (200×) per kidney were skeletonized, followed by quantification of their branching and branch length using Fiji.

Data visualization was performed using GraphPad Prism (v9.0.0; GraphPad Software Inc., La Jolla, CA) and R software with the tidyverse^57^, ggpubr^58^, Raincloud plot^59^ packages, and Rstudio^60^.

### Western blot analysis

For Western blotting, cells were lysed with RIPA buffer (Santa Cruz Biotechnology, Dallas, TX) to isolate the proteins. Then, the samples were reduced with 50 mM dithiothreitol (Invitrogen) for 10 min at 90 °C, followed by SDS-PAGE and transfer to porous polyvinylidene fluoride (PVDF) membranes (Merck Millipore, Burlington, MA). The membranes were blocked in 5% milk in 0.1% TBST and incubated overnight with the primary antibodies, including rabbit polyclonal anti-α-SMA (1:1000; #19245; Cell Signaling Technology, Danvers, MA), rabbit polyclonal anti-Vimentin (1:1000; #5741; Cell Signaling Technology), rabbit polyclonal anti-PAI-1 (1:1000; #27535; Cell Signaling Technology), rabbit polyclonal anti-Col1a1 (1:1000; #91144; Cell Signaling Technology, Danvers, MA), and anti-β-actin (1:3000; #4967; Cell Signaling Technology). Next, the membranes were incubated with horseradish peroxidase (HRP)-conjugated secondary antibodies (Cell Signaling Technology), followed by treatment with ImmunoStar LD (Fujifilm Wako, Osaka, Japan), an enhanced chemiluminescence buffer, and imaging with ChemiDoc MP Imaging System (Bio-Rad, Hercules, CA).

### Statistics

Data are presented as the mean ± standard error of mean (SEM) or median (interquartile range) as appropriate. Differences between two groups were compared using the unpaired *t*-test with Welch’s correction. For multiple comparisons, we performed one-way ANOVA followed by Tukey’s multiple comparisons test or Sidak’s multiple comparisons test. Pearson’s correlation coefficient was used to analyze statistical relationships between two variables. For survival analyses, Kaplan–Meier plots were drawn, and statistical differences were evaluated using log-rank tests. For multivariate analysis, the Cox proportional hazards model was used. The Schoenfeld residue was used to check proportional hazards. Statistical analyses were performed using GraphPad Prism v9 and R software with the tidyverse^57^, ggpubr^58^, survminer^61^, survival^62^, gtsummary^63^, flextable^64^ packages and Rstudio^60^. *p*-values < 0.05 were considered significant.

## Results

### Meflin marks a PMC subset in the kidneys

**Figure 1:**
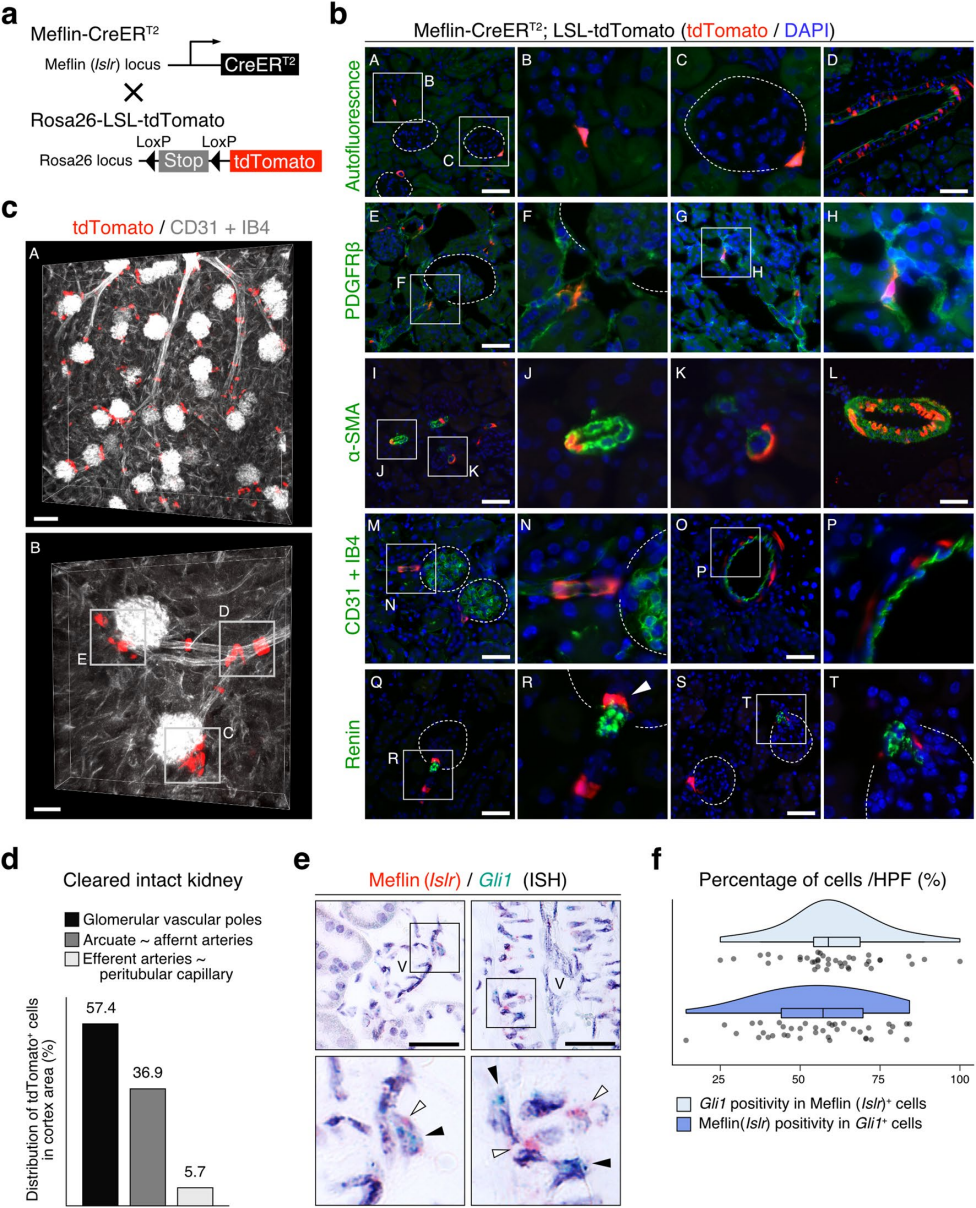
Distribution of Meflin^+^ cells in the normal kidney. (**a**) Meflin-CreER^T2^ mice were crossed with Rosa26-LSL-tdTomato mice, followed by TAM administration (i.p., 0.1 mg/g; 10 times) to evaluate Meflin^+^ (tdTomato^+^) cells. (**b**) Frozen kidney tissue sections prepared from Meflin-CreER^T2^; LSL-tdTomato mice were stained for PDGFRβ, ɑ-SMA, CD31, IB4, and renin (green). Circles surrounded by dotted lines indicate glomeruli. White arrowhead in **R** indicates tdTomato^+^/renin^+^ cells. Scale bar, 50 µm. (**c**) Kidney tissue clearing of kidney of Meflin-CreER^T2^; LSL-tdTomato mice showed Meflin^+^ cells surrounding the vasculatures. Scale bar, 80 (**A**) and 40 (**B**) µm. Boxed regions (**C–E**) indicate tdTomato^+^ cells around glomerular vascular poles, afferent arteries, and efferent arteries, respectively. (**d**) Distribution of tdTomato^+^ cells (244 cells) in the cleared renal cortex was evaluated, followed by quantification. (**e,f**) Adult mouse kidney sections were double stained for Meflin (*Islr*, red) and *Gli1* (green) by duplex ISH. White and black arrowheads indicate Meflin (*Islr*) and *Gli1* signals, respectively. Boxed areas were magnified in lower panels. Scale bar, 50 µm. V, small vessels. Raincloud plots (**f**) showing the percentage of *Gli1*^+^ cells in all Meflin (*Islr*)^+^ cells (upper plot) and Meflin (*Islr*)^+^ cells in all *Gli1*^+^ cells (lower plot). A total of 39 high-power fields (400×) from three samples were evaluated. Data were analyzed and visualized using R software^47^.

We first evaluated the localization of Meflin^+^ cells in the kidneys using Meflin-CreERT2; Rosa26-LSL (*loxP*-stop-*loxP*)-tdTomato (hereafter referred to as LSL-tdTomato) mice, which we previously generated (Fig. 1a)^32^. Analysis of renal sections from adult Meflin-CreER^T2^; LSL-tdTomato mice obtained immediately after TAM administration showed that tdTomato^+^ cells, representing Meflin-expressing cells at the time of TAM treatment, were detected in the interstitium area (Fig. 1b(A–D)). Most (93.3 ± 0.83%) of tdTomato^+^ cells located around small vessels were positive for PDGFRβ, a pericyte marker, and their morphology resembled that of classic pericytes (Fig. 1b(E–H), Fig. S2). Although these tdTomato^+^ cells were either positive or negative for α-SMA, another pericyte marker, they were consistently negative for the endothelial cell markers CD31 and isolectin B4 (IB4) (Fig. 1b(I–P)). Notably, tdTomato^+^/α-SMA^+^ double-positive cells were also observed in the media of middle- to large-sized vessels in a mosaic-like manner, but their distribution appeared distinct from that of typical medial smooth muscle cells that were found throughout the media (Fig. 1b(D,I–L)). tdTomato expression was also detected in cells that are closely associated with the glomerular vascular poles (Fig. 1b(A,C,Q–T)). Most tdTomato^+^ cells were negative for renin but a few were renin positive (Fig. 1b(R)). In all analyses, tdTomato expression was not identified in epithelial, endothelial, immune, or blood cells.

The specific expression of Meflin in interstitial cells was confirmed using a publicly available GEO dataset of gene expression profiles of various lineage cells in the mouse kidneys (accession number: GSE52004; Fig. S3a)^44^. Meflin (*Islr*) mRNA was detected only in the lineage of interstitial forkhead box D1 (Foxd1)^+^ cells but not in those of endothelial cells, macrophages, or renal tubular epithelial cells. The perivascular localization of tdTomato^+^ cells was further corroborated by optical imaging of a cleared intact kidneys from TAM-administered Meflin-CreER^T2^; LSL-tdTomato mice (Fig. 1c,d, Movie S1). We found that tdTomato was mostly expressed in cells localized near the glomerular vascular poles and wrapped around arcuate and afferent arteries. A limited population of tdTomato^+^ cells were observed around efferent arteries and peritubular capillaries. Meflin expression was further examined by ISH, that confirmed the localization of Meflin^+^ cells in the perivascular areas of adult mouse and human kidneys (Fig. S3b,c). Taken together, these data clearly indicate that Meflin marks at least some subset(s) of PMCs in the kidneys.

### Gene expression profile of Meflin^+^ PMCs overlaps with that of classic pericytes

**Figure 2:**
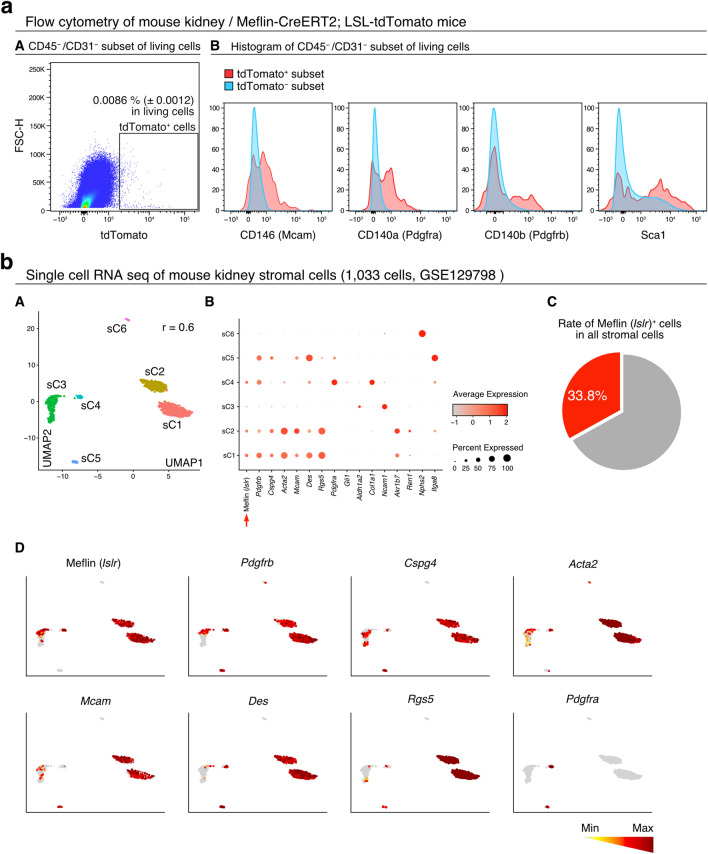
Expression of Meflin in cells positive for conventional pericyte or PMC markers. (**a**) Flow cytometry of cells isolated from the kidneys of adult Meflin-CreER^T2^; LSL-tdTomato mice administered TAM (*n* = 3); 0.0086 ± 0.0012% of CD45^−^/CD31^−^ living cells were positive for tdTomato (**A**). Histogram of tdTomato^+^ (red) and tdTomato^−^ (blue) subsets stained with typical pericyte markers, including CD146 (Mcam), CD140a (Pdgfrα), CD140b (Pdgfrβ), and Sca1 (**B**). (**b**) UMAP plots showing distinct stromal cell populations (sC1–6) identified by single-cell RNA-seq of all stromal cells isolated from the mouse kidney (GEO accession number GSE129798) (**A**). r indicates the clustering resolution. Meflin (*Islr*) mRNA (indicated with an arrow) was expressed in the stromal cell subsets sC1, sC2, and sC4, overlapping with other stromal cell markers to varying degrees (**B**). Meflin was expressed in 33.8% of all stromal cells (**C**). Feature plots showing the expression of Meflin and other pericyte and PMC markers in the stromal cell subsets (**D**). Data were analyzed and visualized using R software^47^.

To further characterize Meflin^+^ PMCs, we double-stained tissue sections from an adult mouse kidney for Meflin and other PMC markers by ISH (Fig. 1e,f, Fig. S4). Most Meflin^+^ PMCs were positive for the pericyte markers *Pdgfrb*, *Pdgfra*, *Cspg4*, and *Acta2* (the gene encoding α-SMA). Approximately 50–60% of Gli1^+^ PMCs, that are the origin of myofibroblasts responsible for fibrosis in renal diseases^21^, were positive for Meflin (Fig. 1e,f). Flow cytometry analysis of all cells isolated from the kidneys of TAM-administered adult Meflin-CreER^T2^; LSL-tdTomato mice revealed that tdTomato^+^ cells constituted less than 0.01% of all kidney cells and were contained in the CD31^−^/CD45^−^ cell fraction (Fig. S5). These CD31^−^/CD45^−^/tdTomato^+^ cells were positive for the pericyte markers MCAM, PDGFRα, and PDGFRβ as well as the fibroblast marker Sca1, confirming our histological analysis (Fig. 2a). Meflin was not co-expressed with *Epo*, a marker of renal interstitial fibroblasts^4,65^, indicating that Meflin^+^ PMCs are similar to pericytes (Fig. S4).

We next validated the gene expression profile of Meflin^+^ cells using the single-cell RNA-seq dataset of an adult mouse kidney (GSE129798)^49^. Meflin mRNA was detected in 1.19% of total kidney cells (373/31,258 total cells), and a uniform manifold approximation and projection (UMAP) representation of single-cell transcriptome profiles showed that Meflin was specifically detected in stromal cell clusters (Str1–3; Fig. S6). Sub-clustering of these clusters (sC1–6) revealed that the majority of Meflin^+^ PMCs, which constitute 33.8% of all stromal cells, were included in subclusters sC1, 2, and 4 that express *Pdgfrb*, *Cspg4*, *Acta2*, *Mcam*, *Des* (the gene encoding desmin), and *Rgs5* mRNA (Fig. 2b). These results indicate that Meflin^+^ PMCs share multiple markers with conventional pericytes in the normal mouse kidneys.

### Meflin^+^ PMCs possess renin-producing capacity

**Figure 3:**
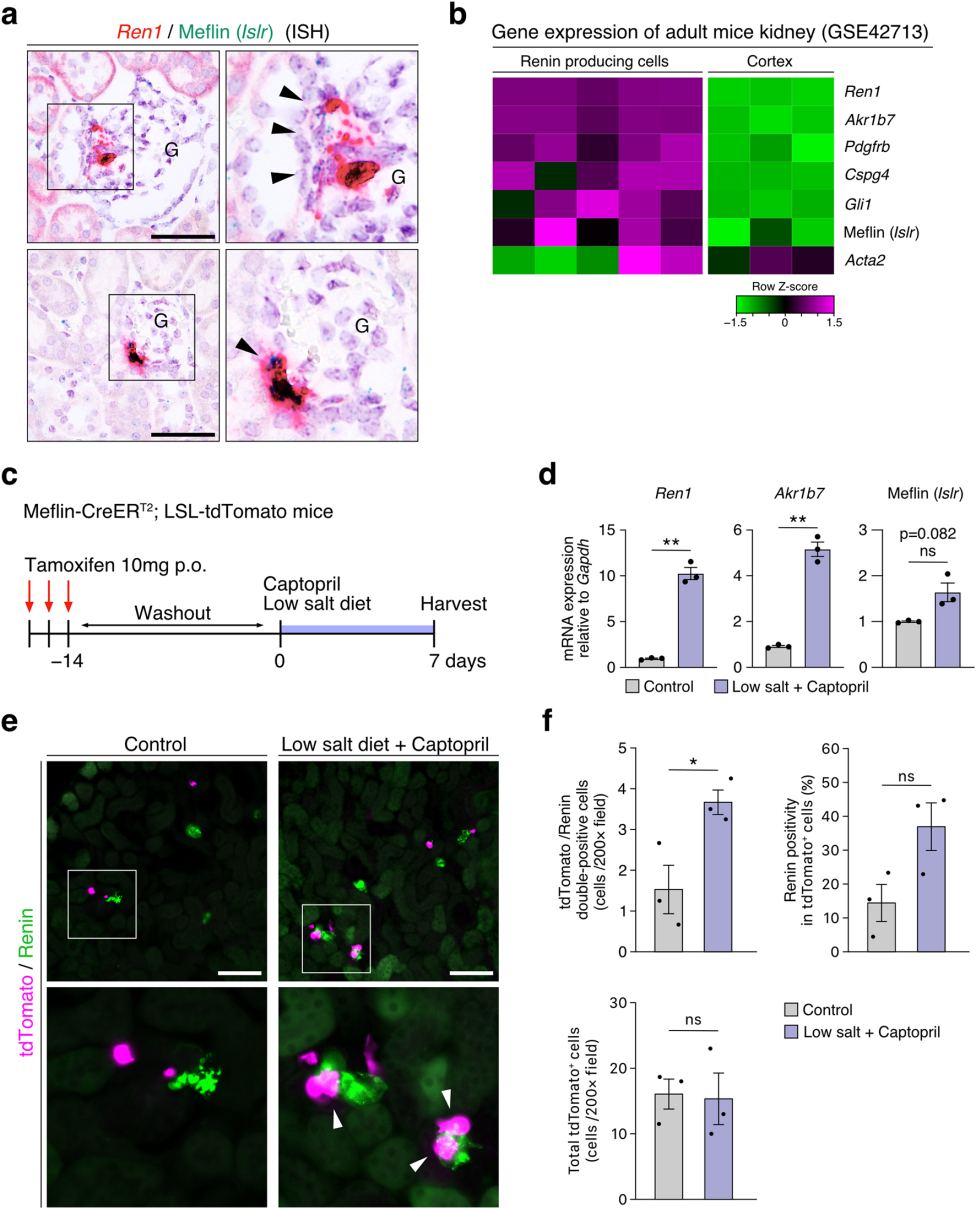
Meflin^+^ PMCs constitute a population of renin-producing cells in the kidneys. (**a**) Adult mouse kidney sections were double stained for renin (*Ren1*, red) and Meflin (*Islr*, green) by duplex ISH. Arrowheads indicate renin and Meflin double-positive cells. Boxed areas were magnified in adjacent panels. Scale bar, 50 µm. G, glomerulus. (**b**) Gene expression profiles of renal cortex cells of a renin reporter mouse line after single cell amplification (microarray database, GSE42713). Expression of Meflin, renin, and other PMC markers in five single-cell amplified renin-producing cells (left) and three non-renin-producing cells (right) isolated from the cortex of the adult renin reporter mice. Data were analyzed using GEO2R and visualized using R software^47^. (**c**) Meflin-CreER^T2^; LSL-tdTomato mice administered TAM were fed captopril (0.5 g/L in drinking water) and 0.02% NaCl diet (low-salt diet) for 7 days after a 14-day washout period (*n* = 3/group). (**d**) qPCR of *Ren1*, *Akr1b7*, Meflin (*Islr*) expression in control Meflin-CreER^T2^; LSL-tdTomato mice and those fed captopril and low-salt diet. (**e**) Kidney sections prepared from control Meflin-CreER^T2^; LSL-tdTomato and those fed captopril and low-salt diet were stained for renin. Boxed areas were magnified in lower panels. Scale bar, 100 µm. (**f**) The numbers of tdTomato and renin double-positive cells (upper left) and total tdTomato^+^ cells (lower left), and the ratio of Renin^+^ cells to tdTomato^+^ cells (upper right) in the indicated groups were shown. Three or four fields (200×) per mice obtained from three kidneys in the control and treatment groups, respectively, were evaluated. Data were analyzed and visualized using R software^47^. Bar plots indicate the mean ± SEM. *p < 0.05, **p < 0.01, ***p < 0.001 (unpaired *t*-test with Welch’s correction).

Given the co-expression of renin and Meflin in kidney periglomerular cells (Fig. 1b(Q–T)), we next investigated renin (*Ren1*) and Meflin expression by ISH (Fig. 3a). We found that a small number of cells (14.4 ± 5.48%) located near the glomerular vascular poles co-expressed *Ren1* and Meflin (*Islr*) mRNA. Moreover, publicly available microarray data on renin-expressing cells in mice (GSE42713)^45^ showed that Meflin expression was enriched in renin-positive juxtaglomerular (JG) cells that were also positive for other pericytes markers (Fig. 3b). This was further corroborated by a lineage-tracing experiment using Meflin-CreERT2; LSL-tdTomato mice administered a low-salt diet and captopril for inducing renin production (Fig. 3c–f); quantitative PCR (qPCR) of total kidney mRNA showed that this induction of *Ren1* was accompanied by the upregulation of aldo–keto reductase family 1 member 7 (*Akr1b7*), a known marker of renin-producing JG cells. Although not statistically significant, Meflin expression also tended to be upregulated by the low-salt diet and captopril treatment (Fig. 3d). Moreover, immunofluorescence (IF) staining showed that a significantly high number of tdTomato^+^ cells were positive for renin in the treatment group, despite the fact that there was no difference in the total number of tdTomato^+^ cells (Fig. 3e,f). The findings suggest that Meflin^+^ PMCs have the potential to induce renin production under hypovolemic conditions and thus participate in renal homeostasis.

### Meflin^+^ PMCs play a pivotal role in maintaining vasculature integrity in the kidneys

**Figure 4:**
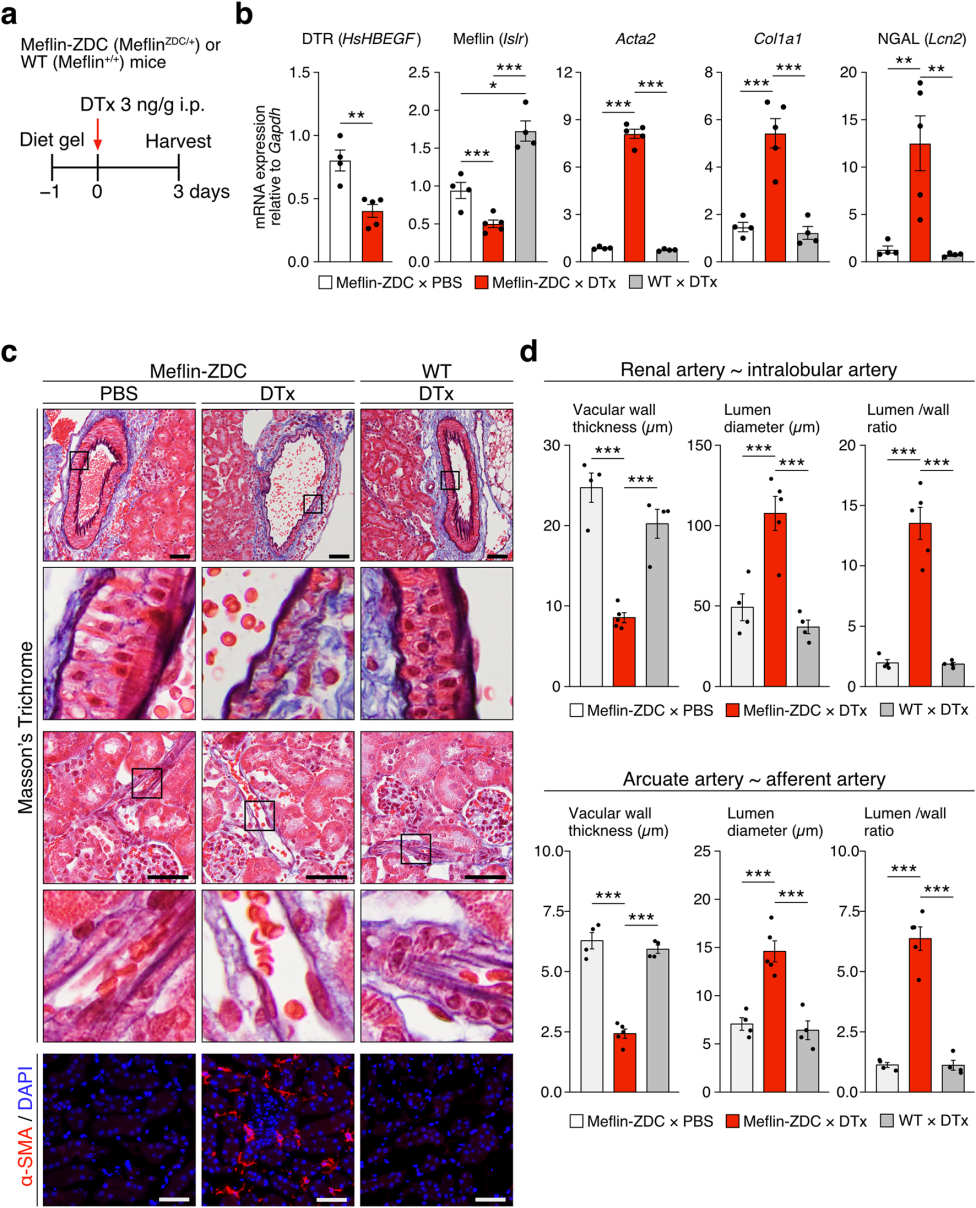
Effects of genetic ablation of Meflin^+^ cells on the normal kidneys. (**a**) Meflin-ZDC mice treated with either PBS (*n* = 4) or DTx (3 ng/g; *n* = 5) and WT mice treated with DTx (*n* = 4) were sacrificed three days after DTx administration, followed by removal of the kidneys for analyses. These mice were fed with Diet gel to reduce gastrointestinal toxicity caused by the ablation of Meflin^+^ cells in the intestine. (**b**) qPCR of human DTR (encoded by *HsHBEGF*), Meflin (*Islr*), α-SMA (*Acta2*), *Ngal*, and *Col1a1* in the indicated groups. (**c**) Masson’s trichrome staining of the harvested kidneys showed dilated vasculatures in DTx-treated Meflin-ZDC mice (top 4 panels). IF staining for α-SMA revealed significant proliferation of ɑ-SMA^+^ cells in the interstitium of Meflin-ZDC mice treated with DTx (bottom panel). Boxed areas were magnified in respective lower panels. Scale bar, 50 µm. (**d**) Measurement and quantification of the thickness, diameter, and lumen/wall ratio of renal and intralobular (upper panel) and arcuate and afferent (lower panel) arteries of the indicated groups. For each group, 5 cross-sections of vasculature per sample (upper panel) and 10 cross-sections of vasculature per sample (lower panel) were evaluated. Data were analyzed and visualized using R software^47^. Bar plots indicate the mean ± SEM. *p < 0.05, **p < 0.01, ***p < 0.001 (unpaired *t*-test with Welch’s correction for *HsHBEGF* in (**b**); one-way ANOVA followed by Tukey’s multiple comparisons test for *Islr, Acta2, Col1a1*, and *Ngal* in (**b) and all the parameters in (d**)).

To investigate the function of Meflin^+^ PMCs in the normal kidneys, we examined changes after genetic ablation of Meflin^+^ PMCs. To this end, we adopted Meflin-ZDC mice that heterozygously express the ZDC [ZsGreen, DTR, and Cre recombinase) cassette under the Meflin gene promoter with each sequence separated by the ribosomal skip T2A sequence^32^. Three days after i.p. injecting Meflin-ZDC mice with DTx, expression levels of Meflin and the human heparin binding epidermal growth factor (*HsHBEGF*) gene, which encodes DTR, decreased by almost half (53%) compared with PBS-treated Meflin-ZDC mice (Fig. 4a,b). Consistent with this, the number of Meflin^+^ cells decreased from 50 ± 1 cells/high-power field (HPF) in the kidney sections of PBS-treated Meflin-DTR mice to 10 ± 2 cells/HPF in those of DTx-treated Meflin-DTR mice, showing 79.8% reduction in Meflin^+^ cell number after a single dose of DTx (Fig. S7a,b). Concomitantly, DTx-treated mice exhibited significantly decreased media thickness of small-sized glomerular afferent and medium-sized intra-lobular arteries and increased lumen diameters compared with that of PBS-treated mice (Fig. 4c,d). This suggests that Meflin^+^ PMCs play important roles in maintaining vascular structure or function. Transmission electron microscopy also revealed dilated peritubular capillaries in DTx-treated mice (Fig. S8).

Intriguingly, the ablation of Meflin^+^ PMCs increased the expression of neutrophil gelatinase-associated lipocalin (Ngal), a marker of renal tubular injury^66^ (Fig. 4b). This suggests that the loss of Meflin^+^ PMCs or subsequent dilatation of peritubular vasculature leads to the dysfunction or degeneration of tubular epithelial cells. This was consistent with a previous report that showed that ablation of Gli1^+^ PMCs induced mild tubular injury^67^. Furthermore, we found that genes encoding α-SMA and Col1a1 were significantly upregulated in DTx-treated Meflin-ZDC mice, suggesting the initiation of a reparative or fibrotic process by the loss of Meflin^+^ PMCs (Fig. 4b). IF staining showed the proliferation of α-SMA^+^ cells, which were also positive for PDGFRβ and *Col1a1* and probably represented myofibroblasts, in the interstitial area of DTx-treated mice (Fig. 4c; lower panel, Fig. S9). Although the total number of *Gli1*^+^ cells, which are reportedly known to give rise to α-SMA^+^ myofibroblasts, was unchanged, that of *Gli1*^+^Meflin (*Islr*)^−^ cells significantly increased in DTx-treated Meflin-ZDC mice, being accompanied by an increase in *Gli1* expression level (Fig. S10). These changes in the vasculature and myofibroblast proliferation were most noticeable at 3 days after DTx injection, followed by recovery by day 6 (Fig. S11a–c). Notably, this recovery was accompanied by an increase and decrease in Meflin and α-SMA expression, respectively (Fig. S11b,c). The findings suggest that the remaining Meflin^+^ PMCs that were not ablated by DTx treatment undergo compensatory or reparative proliferation in response to vasculature degeneration. Therefore, Meflin^+^ PMCs appear to play an essential role in maintaining the integrity of vessel and renal tubule functions and suppressing the proliferation of α-SMA^+^ cells, the ablation of which results in the proliferation of α-SMA^+^ fibroblasts that do not originate from Meflin^+^ PMCs.

### Meflin^+^ PMCs proliferate and give rise to heterogeneous fibroblast populations during renal fibrosis

**Figure 5:**
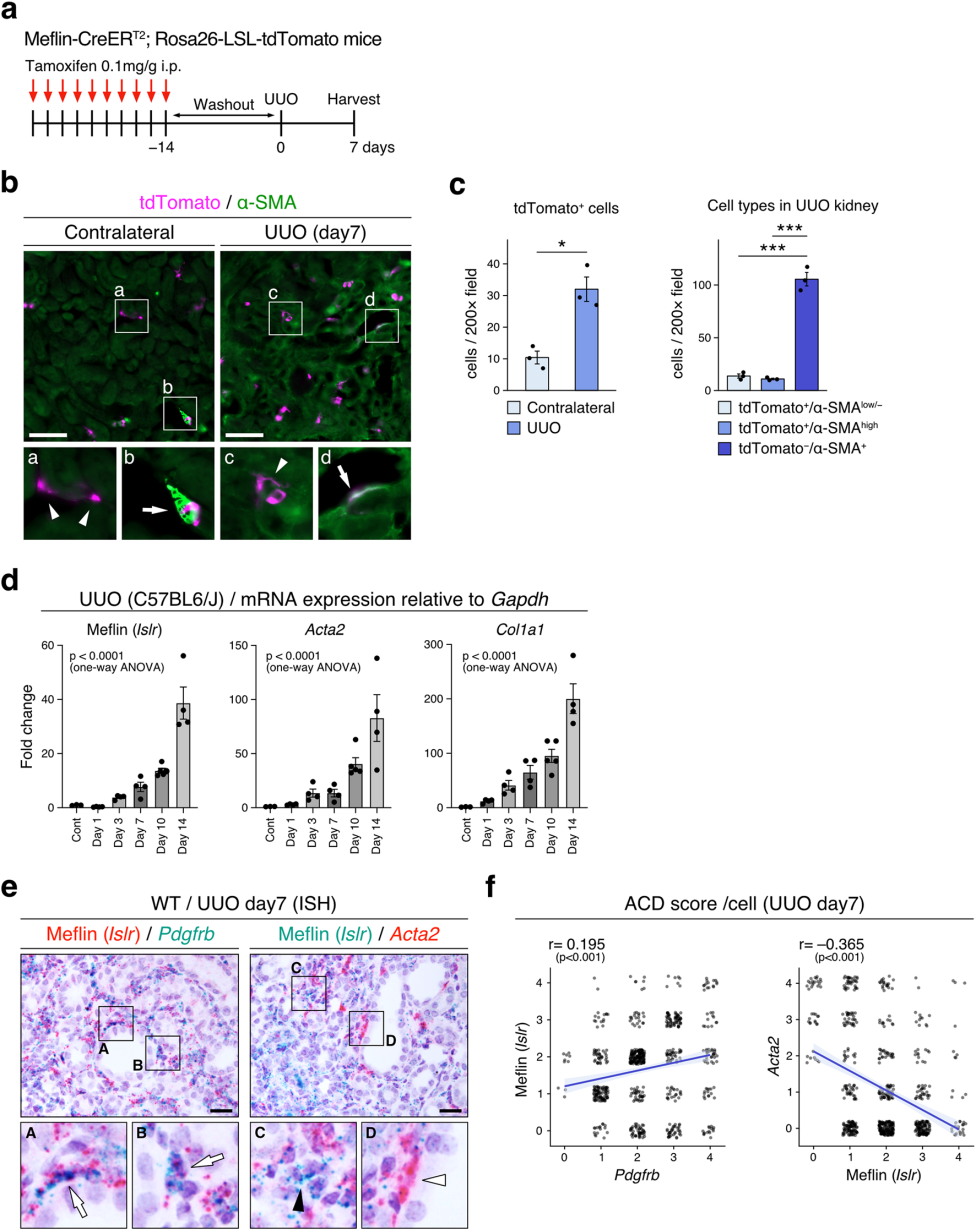
Meflin^+^ PMCs proliferate and give rise to heterogeneous fibroblast populations in a renal fibrosis mouse model. (**a**) Meflin-CreER^T2^; LSL-tdTomato mice were subjected to UUO surgery 14 days after i.p. TAM-administration (10 times). (**b**) Tissue sections prepared from UUO and contralateral kidneys of TAM-administered Meflin-CreER^T2^; LSL-tdTomato mice were stained for α-SMA by IF. Arrowheads and arrows indicate tdTomato^+^/α-SMA^low/−^ cells and tdTomato^+^/α-SMA^high^ cells, respectively. (**c**) The numbers of tdTomato^+^ cells in UUO (day 7) and contralateral kidneys were counted, followed by quantification (left). The α-SMA^+^ cells were classified based on the expression of tdTomato and the degrees of α-SMA expression (right). For each group, 5 fields (200×) per sample obtained from three kidneys were evaluated. Data were analyzed and visualized using R software^47^. Bar plots indicate the mean ± SEM. (**d**) qPCR of time-dependent changes in Meflin (*Islr*), *Acta2*, and *Col1a1* in the UUO kidneys of WT mice (*n* = 3–5/group). (**e**) The kidney of WT mice 7 days after UUO induction were stained for Meflin (*Islr*), *Pdgfrb*, and *Acta2* by duplex ISH. Arrows indicate co-expression of Meflin (*Islr*) and *Pdgfrb*; black and white arrowheads indicate Meflin (*Islr*)^high^/*Acta2*^low^ cells and Meflin (*Islr*)^low^/Acta2^high^ cells, respectively. (**f**) Semi-quantification of Meflin (*Islr*), *Pdgfrb*, and *Acta2* in fibroblasts of the kidney interstitium 7 days after UUO induction. The number of dots per cell was evaluated for all cells, except epithelial cells, in four fields (1000×) using a RNAscope scoring system. Correlation testing were performed Pearson’s correlation coefficient. Data were analyzed and visualized using R software^47^. **p < 0.01, ***p < 0.001 [unpaired *t*-test with Welch’s correction (**c**); one-way ANOVA (**d**)] Scale bars, 100 (**b**) and 20 (**e**) µm.

Next, to examine the behavior of Meflin^+^ PMCs in renal fibrosis, we traced the lineage of Meflin-expressing PMCs by subjecting TAM-administered Meflin-CreER^T2^; LSL-tdTomato mice to UUO (Fig. 5a). Seven days after surgery, a significant proliferation of tdTomato^+^ fibroblastoid cells was observed in the injured kidney compared with the contralateral healthy kidney (Fig. 5b,c; left panel). Consistent with this, qPCR showed a time-dependent increase in Meflin (*Islr*), *Acta2*, and *Col1a1* expression in the UUO kidneys (Fig. 5d). This increase in Meflin expression in the UUO model was further confirmed by ISH that showed that Meflin^+^ PMC proliferation in the fibrotic interstitium (Fig. S12a,b). We also examined the proliferation of Meflin^+^ PMCs in the kidneys of adenine-induced kidney injury^68^, and observed a patchy distribution of injured regions caused by adenine crystals (Fig. S12c). We found that Meflin^+^ PMCs were increased mainly in the injured areas, indicating that Meflin^+^ PMCs proliferate or migrate in response to injury. A publicly available dataset showing changes in gene and protein expressions in the UUO and folic acid-induced kidney injury^46,48^ also demonstrated the upregulation of Meflin in parallel with increased in α-SMA and collagen type I and III expressions (Figs. S12d, S13). These data showed that Meflin^+^ PMCs proliferate upon renal injury, which is in line with a previous notion that PMCs proliferate in various kidney diseases^21,69^.

However, lineage-tracing experiments also revealed that only 9.4 ± 0.6% of all α-SMA^+^ cells were tdTomato^+^ cells despite PMCs being reported as major origins of myofibroblasts, suggesting that Meflin lineage cells are less likely to differentiate into myofibroblasts. In addition, some tdTomato^+^ Meflin lineage cells were highly positive for α-SMA (α-SMA^high^), whereas others were weakly positive or negative (α-SMA^low/−^), indicating a heterogeneity of cells derived from Meflin^+^ PMCs in terms of their differentiation into myofibroblasts (Fig. 5c; right panel). Double ISH staining also demonstrated the heterogeneity of α-SMA expression in Meflin^+^ cells after UUO induction (Fig. 5e). Specifically, we adopted a semi-quantitative scoring system based on counting signal dots in ISH analysis, and found that the majority of Meflin^+^ cells were positive for *Pdgfrb* (98.17%, 535/545 cells), while only a limited number of cells were double-positive for Meflin (*Islr*) and *Acta2* (43.38%, 282/650 cells, Fig. 5f).

The inverse correlation between Meflin and α-SMA expression was also confirmed by publicly available single-cell transcriptomic data obtained from human allograft kidney tissues undergoing clinical rejection, which are accompanied by fibrotic alterations (GSE109564; Fig. S14)^50^. Meflin (*ISLR*) and *ACTA2* were preferentially expressed in the same cell cluster as *PDGFRB*, but further subclustering of the *PDGFRB*^+^ fibroblast cluster revealed that Meflin (*ISLR*) was expressed in fibroblast subsets that are distinct from those expressing *ACTA2* (Fig. S14c). These findings suggest that proliferating Meflin^+^ PMCs or their lineage may constitute unique fibroblast subsets that are different from α-SMA^+^ myofibroblasts in fibrotic renal disease.

### Prognostic value of the number of Meflin^+^ PMCs in human kidney diseases

**Figure 6:**
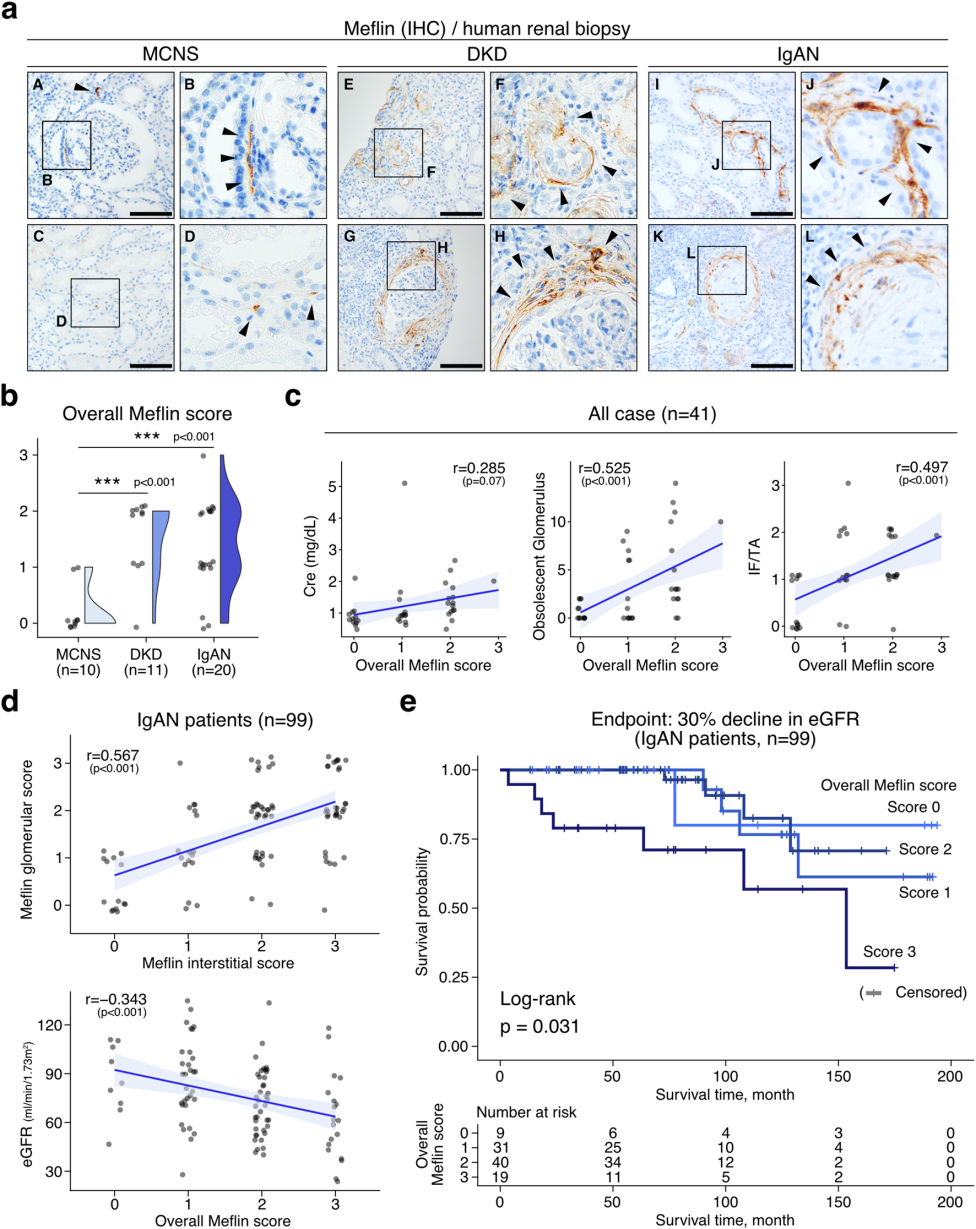
Meflin expression is correlated with poor renal prognosis in patients with chronic kidney diseases. (**a**) Representative Meflin IHC using kidney biopsy samples from patients with MCNS, DKD, and IgAN. Arrowheads indicate Meflin^+^ areas. Scale bar, 50 µm. (**b**) Raincloud plots showing overall Meflin scores in the indicated groups. ***p < 0.001 (one-way ANOVA followed by Tukey’s test). (**c**) Correlations between overall Meflin score and serum Cre, obsolescent glomerulus counts, and IF/TA in all cases of MCNS, DKD, and IgAN (*n* = 41) were analyzed using Pearson’s correlation coefficient. (**d**) Biopsy samples from patients with IgAN (*n* = 99) were analyzed for associations between Meflin glomerular score and Meflin interstitial score (upper panel) and overall Meflin score and eGFR (lower panel), followed by correlation testing using Pearson’s correlation coefficient. (**e**) Kaplan–Meier survival analysis; patients with IgAN and high overall Meflin scores exhibited poor renal prognosis (a 30% decline in eGFR was used as the end point) compared with those with low overall Meflin scores. Log-rank test was used for statistical analysis. Data were analyzed and visualized using R software^47^ in (**b–e**).

Next, we examined the prognostic value of Meflin^+^ PMCs by immunohistochemistry (IHC) of human kidney biopsy samples (Fig. 6). Tissues obtained from patients with MCNS exhibited limited fibrotic changes and limited proliferation of Meflin^+^ PMCs in the periglomerular and interstitial regions. In contrast, there were much more Meflin^+^ PMCs in tissue samples from patients with DKD and IgAN (Table S1, Fig. 6a,b). Consistent with the UUO model findings, the proliferation of Meflin^+^ PMCs was observed in the interstitial fibrotic area and those around or in extraglomerular crescents and obsolescent glomerulus, supporting the notion that Meflin^+^ PMCs proliferate in response to renal injury and fibrosis. The infiltration of Meflin^+^ PMCs was also significantly correlated with the presence of obsolescent glomeruli and the degree of fibrosis, which was determined by the interstitial fibrosis/tubular atrophy (IF/TA) scoring system, suggesting that the number of Meflin^+^ PMCs reflect the activity of kidney diseases (Fig. 6c).

We next examined the impact of Meflin expression on the long-term renal prognosis of IgAN (Table S2). Biopsy samples obtained from 99 patients with IgAN demonstrated significant correlations between Meflin^+^ PMC proliferation in the interstitial fibrotic area and around the glomeruli, and between estimated glomerular filtration rate (eGFR) and overall proliferation of Meflin^+^ PMC, further supporting the possibility that the proliferation of Meflin^+^ PMCs is a surrogate marker of IgAN disease activity (Fig. 6d). Survival analysis, where a 30% decline in eGFR was used as an end point, indicated that the group with the highest Meflin score had the worst renal prognosis during a median follow-up period of 6.2 (4.1–9.6) years (Fig. 6e). The Cox proportional hazards models showed that the proliferation of Meflin^+^ PMCs was a strong independent renal prognostic factor after adjusting for age, sex, eGFR, proteinuria, and tissue fibrosis (MEST-T score) (Table 1).

**Table 1:** Cox proportional hazards models for 99 patients with IgAN.

Characteristics	Univariate			Multivariate								
				Model 1			Model 2			Model 3		
	HR	95% CI	*p*-value	HR	95% CI	*p*-value	HR	95% CI	*p*-value	HR	95% CI	*p*-value
**Meflin expression**^A^												
Meflin low (*n* = 80)	–	–		–	–		–	–		–	–	
Meflin high (*n* = 19)	3.98	1.48, 10.7	**0.006**	5	1.76, 14.2	**0.003**	5.3	1.78, 15.8	**0.003**	4.29	1.45, 12.7	**0.009**
**Age**, per 10 years	1.4	1.01, 1.95	**0.046**	1.32	0.97, 1.80	0.075	0.99	0.63, 1.55	> 0.9	1.1	0.65, 1.83	0.7
**Sex**												
Male (*n* = 43)	–	–		–	–		–	–		–	–	
Female (*n* = 56)	0.32	0.12, 0.90	**0.03**	0.29	0.10, 0.84	**0.022**	0.27	0.09, 0.83	**0.022**	0.29	0.10, 0.87	**0.027**
**eGFR**, per 10 mL/min/1.73 m^2^	0.72	0.55, 0.94	**0.016**				0.77	0.56, 1.05	0.1	0.89	0.63, 1.26	0.5
**Proteinuria**, per 1 g/day	1.23	0.75, 2.01	0.4				1.36	0.77, 2.43	0.3	1.08	0.56, 2.06	0.8
**MEST-T score**												
Score 0 (*n* = 81)	–	–								–	–	
Score 1 (*n* = 11)	1.64	0.36, 7.42	0.5							2.05	0.36, 11.7	0.4
Score 2 (*n* = 4)	10.1	2.71, 37.7	** < 0.001**							6.83	1.09, 42.9	**0.04**

*HR* hazard ratio, *CI* confidence interval, *eGFR* estimated glomerular filtration rate.

^A^Meflin low, Overall Meflin score = 0, 1, 2; Meflin high, Overall Meflin score = 3.

Significance values are given in bold.

### Involvement of Meflin^+^ PMCs in collagenogenesis and fibrosis in the UUO model

**Figure 7:**
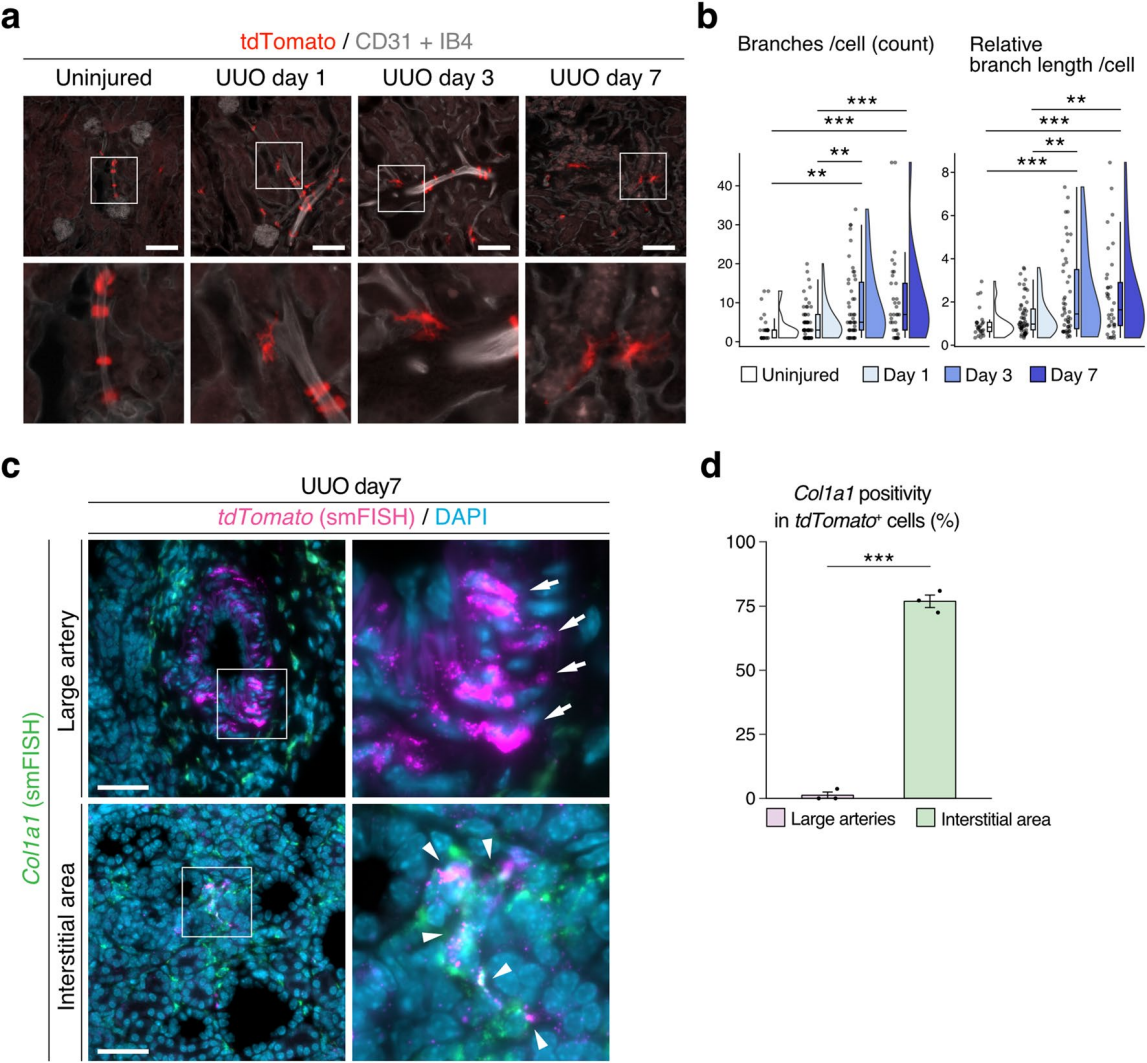
Meflin^+^ PMCs detach from the vasculature and produce collagen during renal fibrosis. (**a,b**) Meflin-CreER^T2^; LSL-tdTomato mice were subjected to UUO surgery 14 days after TAM-administration, followed by staining of uninjured and UUO kidneys for CD31 and IB4 to visualize the vasculature (white). Note that tdTomato^+^ cells surrounding the vasculature in uninjured kidneys were detached from the vasculature in UUO kidneys (**a**). Quantification of morphological changes (branching and branch length) of tdTomato^+^ cells (total 167 cells) over time after UUO surgery (**b**). (**c,d**) smFISH analysis for *tdTomato* and *Col1a1* (UUO day 7). Although *tdTomato*^+^ cells surrounding the vasculature do not express *Col1a1*, *tdTomato*^+^ cells detached from the vasculature in interstitial area express *Col1a1* (**c**). Shown in (**d**) is the quantification of *Col1a1* positivity in *tdTomato*^+^ cells. For each group, 3 or 4 cross-section (400×) of the vasculature per sample and 5 sections of the interstitial area (400×) per sample obtained from three kidneys were evaluated. **p < 0.01, ***p < 0.001 [one-way ANOVA followed by Tukey’s multiple comparisons test (**b**); unpaired *t*-test with Welch’s correction (**d**)] Boxed areas were magnified in adjacent panels. Scale bars, 100 (**a**) and 50 (**c**) µm. Data were analyzed and visualized using R software^47^ in (**b,d**).

We next tried to address the function of proliferating Meflin^+^ PMCs in the UUO model. Interestingly, tdTomato^+^ cells were detached from the vasculature and began exhibiting dendritic morphology with many fine branches as UUO induction progressed, suggesting some changes in cellular function may have occurred under this condition (Fig. 7a,b). Indeed, smFISH analysis for *Col1a1* revealed that only tdTomato^+^ cells detached from the vasculature, not tdTomato^+^ cells surrounding the vasculature, express *Col1a1* (Fig. 7c,d)*.*

**Figure 8:**
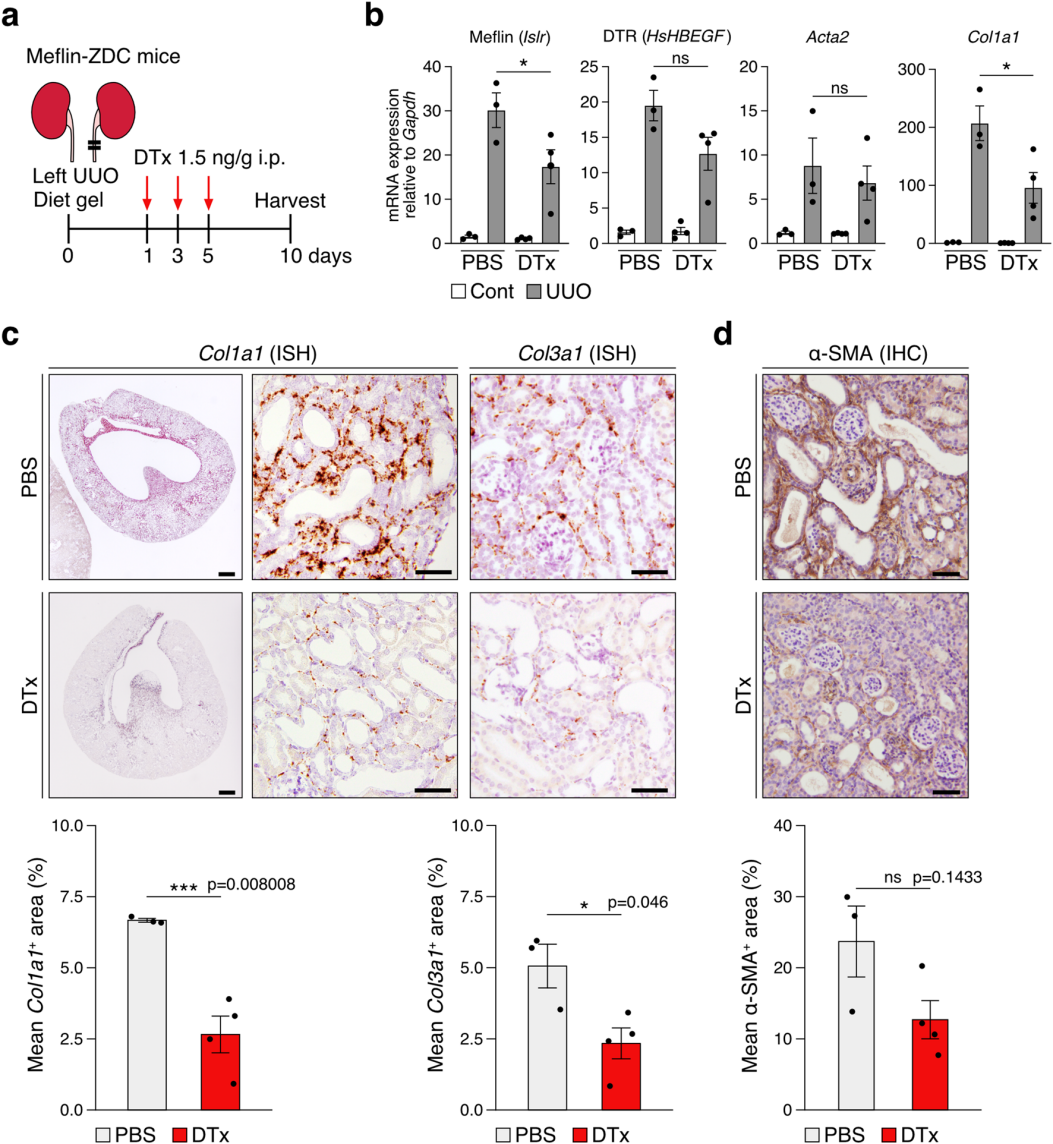
Effect of genetic ablation of Meflin^+^ PMCs on collagen production and fibrosis in the UUO model. (**a**) Meflin-ZDC mice were i.p. injected with DTx (1.5 ng/g; *n* = 4) or PBS (*n* = 3) at 1, 3, and 5 days after UUO surgery, followed by kidney harvesting 10 days later. (**b**) qPCR of Meflin (*Islr*), DTR (*HsHBEGF)*, α-SMA (*Acta2*), and *Col1a1* expression in control (PBS) and DTx-treated UUO kidneys. Cont, contralateral. (**c,d**) ISH for *Col1a1*, *Col3a1* (**c**), and IHC for α-SMA (**d**) in tissue sections from control (PBS) and DTx-treated UUO kidneys, followed by quantification of stained areas (%) using ImageJ software. For each group, 6–8 fields (400×) per sample (**c**) and 10–13 fields (400×) per sample (**d**) were evaluated. *p < 0.05, **p < 0.01, ***p < 0.001 (one-way ANOVA followed by Tukey’s multiple comparisons test in (**b**); unpaired *t*-test with Welch’s correction in (**c**,**d**)). Data were analyzed and visualized using R software^47^ in (**c,d**).

To reveal the function of Meflin^+^ PMCs in reparative process or fibrosis, we subjected Meflin-ZDC; LSL-tdTomato mice to UUO, followed by genetic ablation of Meflin^+^ PMCs through i.p. DTx administration. The kidneys were analyzed 10 days after surgery (Fig. 8a), and qPCR showed that Meflin (*Islr*) and DTR (*HsHBEGF*) expression was downregulated in DTx-treated UUO kidneys compared with PBS-treated UUO kidneys (Fig. 8b). Although there were no significant differences in *Acta2* expression*, Col1a1* expression was significantly downregulated in DTx-treated UUO kidneys. ISH and IHC analysis also showed significant reduction of *Col1a1* and *Col3a1* mRNA expression and α-SMA protein expression in DTx-treated UUO kidneys compared with control, further supporting the notion that Meflin^+^ PMCs are involved in reparative collagen synthesis or fibrosis after renal injury (Fig. 8c,d).

### Meflin is transcriptionally downregulated by TGF-β and suppresses TGF-β action on myofibroblast differentiation

**Figure 9:**
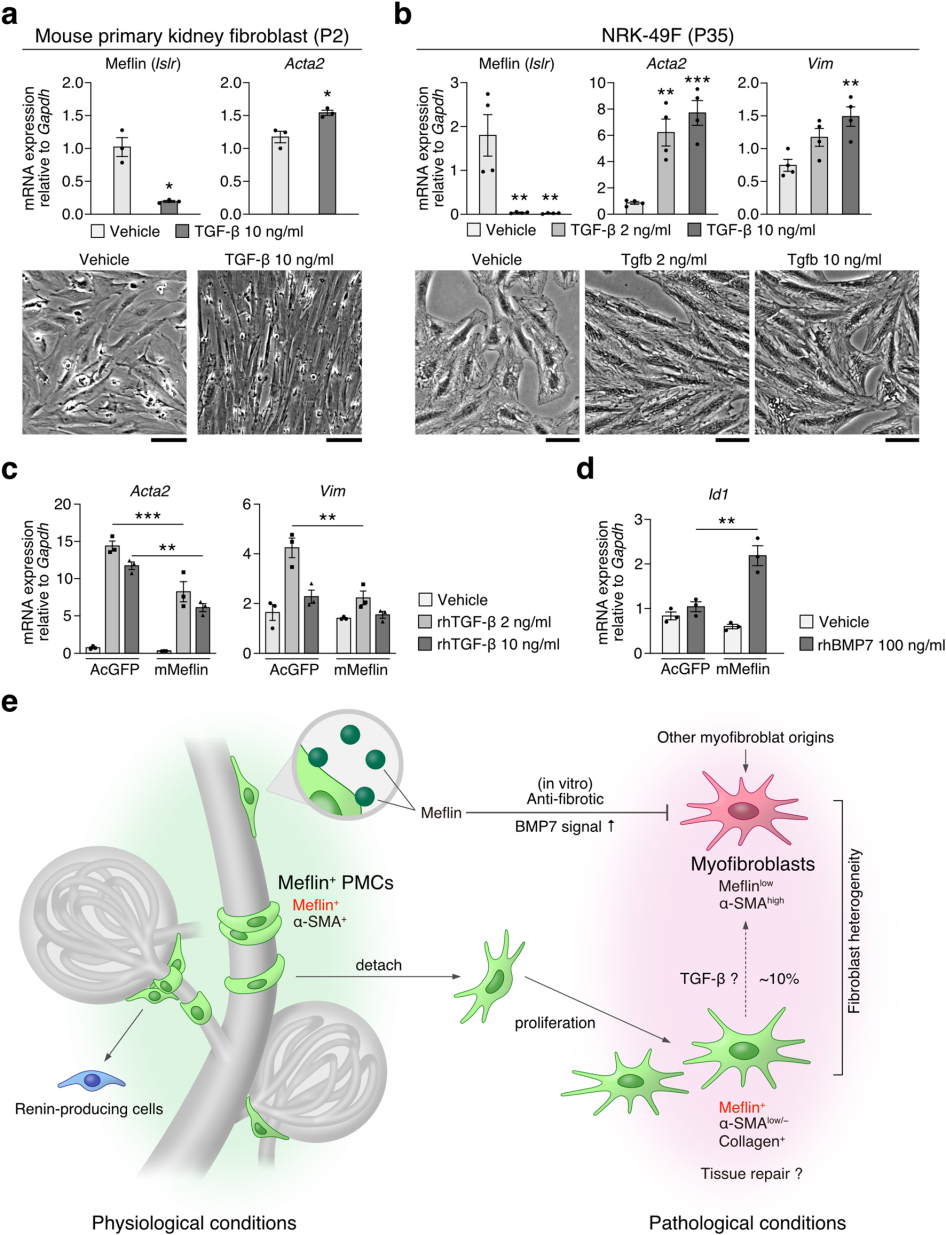
Meflin counteracts TGF-β signaling in kidney fibroblasts. (**a,b**) Primary cultured kidney fibroblasts isolated from C57B6/J mice (*n* = 3/group) (**a**) or the rat kidney fibroblast cell line NRK-49F (*n* = 4/group) (**b**) were stimulated with recombinant TGF-β (2 or 10 ng/mL) for 24 h, followed by qPCR for the indicated genes. Morphology of the TGF-β-treated fibroblasts (lower panels). Scale bars, 50 µm. (**c,d**) NRK-49F cells transduced with AcGFP (control) or mouse Meflin (mMeflin) were stimulated with recombinant human TGF-β (**c**) or recombinant human BMP7, (**d**) for 24 h with indicated concentration, followed by qPCR for the indicated genes (*n* = 3/group). *p < 0.05, **p < 0.01, ***p < 0.001 (unpaired *t*-test with Welch’s correction in (**a**,**b**); one-way ANOVA followed by Tukey’s multiple comparisons test in (**c**,**d**)). (**e**) Schematic illustrating the working hypothesis for the role of Meflin and Meflin^+^ PMCs in the kidneys. Meflin^+^ PMCs reside around the vasculature and glomerular vascular poles and exhibit a morphology indistinguishable from that of classic pericytes. These PMCs are involved in vasculature homeostasis and can produce renin under hypovolemic conditions. Upon tissue injuries that induce inflammation or fibrosis, Meflin^+^ PMCs detach from the vasculature with a dendritic morphology, followed by proliferation. Meflin^+^ PMCs are involved in collagenogenesis, but it remains unknown how they contribute to tissue repair or fibrosis. Lineage tracing experiments showed that some Meflin^+^ PMCs give rise to α-SMA^+^ fibroblasts, which constitute a minor population within all α-SMA^+^ fibroblasts during renal fibrosis. Finally, cell culture experiments showed that Meflin suppresses TGF-β signaling and augments BMP7 signaling, consistent with previous studies that reported that Meflin plays an anti-fibrotic role in cardiac and lung fibrosis.

Finally, we attempted to determine the molecular function of Meflin, a GPI-anchored membrane protein, using cultured renal fibroblasts (Fig. 9). Consistent with previous studies and our data that showed an inverse correlation between Meflin and α-SMA expression (Fig. 5)^30,32^, primary cultured mouse kidney fibroblasts and NRK-49F cells, a rat kidney fibroblast line, stimulated with TGF-β exhibited a significant decrease in Meflin expression (Fig. 9a,b). This was accompanied by an increase in *Acta2* and *Vim* (the gene encoding vimentin) mRNA levels and increased spindle morphology. Interestingly, exogenous overexpression of Meflin significantly suppressed TGF-β-mediated *Acta2, Vim*, and *Serpine1*, but not *Col1a1*, expression with different time dependencies in NRK-49F cells (Fig. 9c, Fig. S15a). Some discrepancies between mRNA and protein expression levels of these fibroblasts markers in terms of the effects of Meflin overexpression were observed in the experiments, implicating the involvement of Meflin in complex machineries that may control both gene expression and protein homeostasis (Fig. S15a,b). We also showed that Meflin has a function to augment BMP7 signaling that is known to counteract TGF-β signaling (Fig. 9d). These findings indicate that Meflin suppresses TGF-β activity as well as the TGF-β-mediated differentiation of NRK-49F cells to myofibroblasts.

## Discussion

In the present study, we identified a new subset of PMCs marked by Meflin expression in the kidneys. Meflin^+^ PMCs were indistinguishable from classic Gli1^+^ or PDGFRβ^+^ pericytes in terms of their localization and distribution as determined by histologic and single-cell transcriptomic analyses of the normal kidneys. However, approximately half of Meflin^+^ PMCs were negative for Gli1, and their genetic ablation resulted in the disruption of renal vascular integrity and fibrogenic response as indicated by the proliferation of α-SMA^+^ and *Gli1*^+^Meflin^−^ myofibroblasts, and *Col1a1* upregulation in the kidneys. Previous studies showed that the loss of Gli1^+^ pericytes or Foxd1 lineage cells does not necessarily result in the same pathology as that induced by Meflin^+^ PMC ablation^67,70^. Furthermore, we found that a population of Meflin^+^ PMCs localized in the glomerular vascular pole possess the potential to differentiate into renin-producing cells under decreased renal perfusion conditions. Taken together, we speculate that Meflin^+^ PMCs represent a renal PMC subset that has not been described previously and is distinct from classic pericytes or PMCs in the kidneys (Fig. 9e).

Another feature of Meflin^+^ PMCs is that they significantly proliferated during renal fibrosis but constituted a different subset of fibroblasts from the typical α-SMA^high^ myofibroblasts (Fig. 9e). This was consistent with our lineage-tracing experiment using Meflin-CreER^T2^; LSL-tdTomato mice, where we showed a limited contribution of Meflin lineage cells to the proliferation of α-SMA^high^ myofibroblasts in renal fibrosis. In addition, our experiments using cultured renal fibroblasts showed that the molecular function of Meflin was the suppression of TGF-β-mediated myofibroblast differentiation. These findings indicate that Meflin^+^ PMCs and their lineage cells are functionally different from conventional myofibroblasts. Consistent with this notion are findings reported in previous studies, where Meflin^+^ fibroblasts were found to suppress cancer progression and fibrosis in pancreatic cancer and cardiac and lung fibrosis, respectively, and were distinct from α-SMA^+^ myofibroblasts that promote disease progression^30,32,36^.

Despite the inverse correlation between Meflin and α-SMA expression in proliferating PMCs/fibroblasts under renal disease conditions, our clinicopathological analysis showed a positive correlation between poor renal prognosis and the number of Meflin^+^ PMCs in IgAN. One possible explanation for this finding is that the primary function of proliferating Meflin^+^ PMCs is tissue repair but not the promotion of fibrosis in the injured kidneys. Indeed, genetic ablation of Meflin^+^ PMCs decreased *Col1a1* and *Col3a1* expression, which is essential for collagen production and tissue restoration. Previous studies including one by our group demonstrated that Meflin KO mice are significantly defective in acute tissue repair after the induction of acute myocardial infarction and intestinal inflammation, and are vulnerable to these diseases^30,39^. Moreover, Meflin KO mice exhibited a significant progression of pancreatic cancer when crossed with an autochthonous pancreatic cancer mouse model, indicating that Meflin^+^ CAFs retard cancer progression and are different from conventional cancer-promoting CAFs^32^. Considering the above findings and the fact that cancers have been described as wounds that do not heal^71^, we theorize that Meflin^+^ PMCs are involved in continuous tissue reparative processes under chronic fibroinflammatory conditions such as fibrotic diseases and cancer.

We recently showed that Meflin interacts with BMP7 and augments its signaling^30,35^. We confirmed this results in present study using renal fibroblasts, supporting anti-fibrotic function of Meflin in kidney. However, our present study does not provide Meflin function in vivo kidney disease model. Further investigations will be needed to clarify essential functions of Meflin in kidney disease models that recapitulate human kidney diseases in the future. Moreover, a recent study has suggested the involvement of Meflin in activation of the Yap1 signaling pathway, which also highlights the unique role of Meflin^+^ PMCs that are distinct from those of conventional renal PMCs^39^.

In summary, our present study identified a new subset of Meflin^+^ renal PMCs, that are functionally and spatially distinct from previously described renal PMCs. They are essential for vasculature homeostasis in the normal kidneys, proliferate to give rise to heterogeneous populations of fibroblasts, and are involved in collagen expression after renal injury. The precise and unique contribution of Meflin protein and Meflin^+^ PMCs to chronic kidney disease should be further investigated in future studies, which will lead to a better understanding of the pathogenesis of tissue repair or fibrosis.

## Supplementary Information

Supplementary Information 1.

Supplementary Information 2.

Supplementary Video 1.

Supplementary Video 2.
