# Supplementary material for: A novel renal perivascular mesenchymal cell subset gives rise to fibroblasts distinct from classic myofibroblasts
Source: Sci Rep. 2022 Mar 30;12:5389. doi: 10.1038/s41598-022-09331-5 (PMC8967907; doi:10.1038/s41598-022-09331-5)
Supplement: Supplementary file 1 — Supplementary Information 1. [file 41598_2022_9331_MOESM1_ESM.pdf]

Supplementary Material

A novel renal perivascular mesenchymal cell subset gives rise to fibroblasts distinct from classic myofibroblasts

Minatoguchi *et al.*

Movie S1 and S2. Three-dimensional distribution of tdTomato$^+$ (Meflin$^+$) cells in the adult mouse kidney revealed by tissue clearing.

Kidney sections (Movie 1: width 789 μm, height 802 μm, depth 280 μm, Movie 2: width 316 μm, height 321 μm, depth 56 μm) obtained from tamoxifen-administered Meflin-CreER^{T2}; LSL-tdTomato mice were stained with anti-CD31 antibody and isolectin B4 (IB4) to visualize the vasculature, followed by tissue clearing and image capture. The minor scale interval for each axis indicates 10 μm.

Figure S1

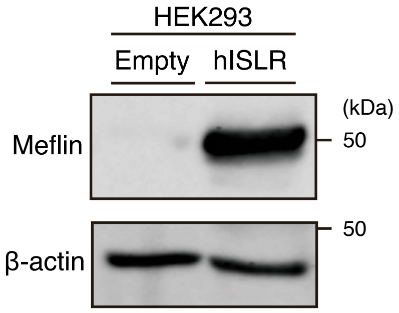

Figure S1. Specificity of anti-Meflin antibody used in the study.

Western blot analysis on lysates isolated from HEK293 cells transfected with either control vector (Empty) or Meflin-expressing vector (hISLR) showed that the rat monoclonal anti-Meflin antibody specifically detected exogenously-expressed Meflin in HEK293 cells. kDa, kilodaltons.

Figure S2

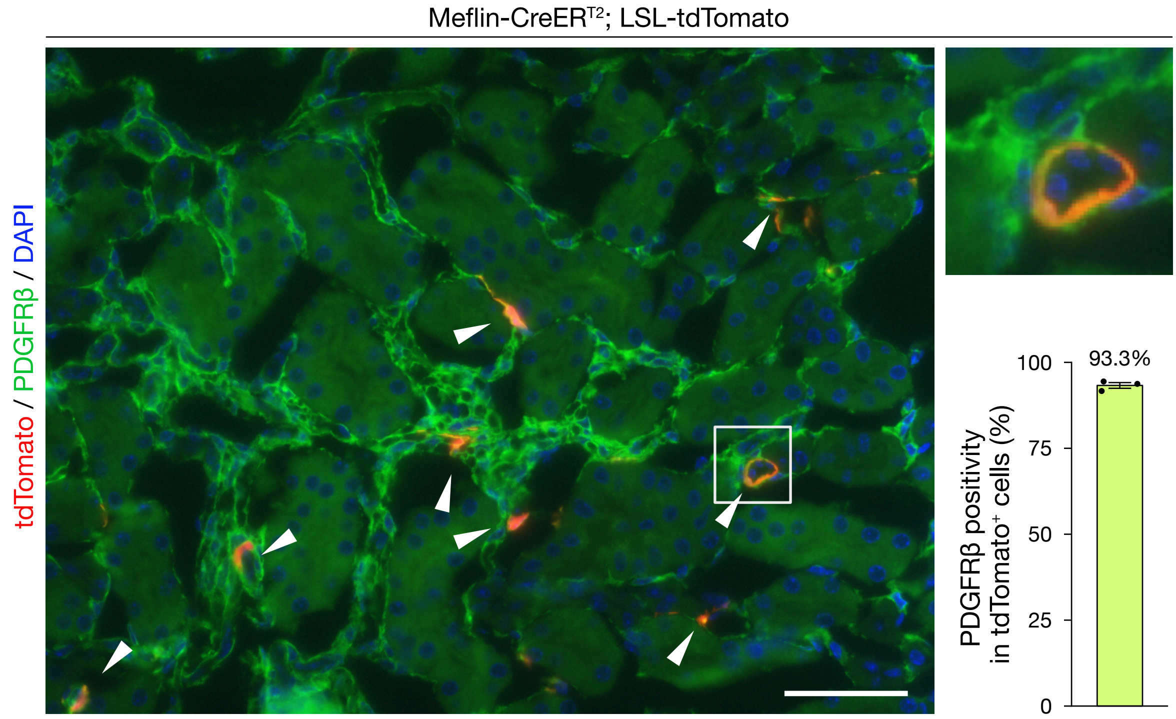

Meflin-CreERT2; LSL-tdTomato
tdTomato / PDGFRβ / DAPI
PDGFRβ positivity
in tdTomato+ cells (%)
100
75
50
25
0
93.3%

Figure S2. Expression of PDGFR β in tdTomato^{+} cells in the kidney of Meflin-CreER^{T2}; LSL-tdTomato mice administered TAM.

Frozen sections prepared from the renal cortex of TAM-administrated Meflin-CreER^{T2}; LSL-tdTomato mice were stained for PDGFRβ by IF (green). PDGFRβ positivity in all tdTomato^{+} cells was quantified in eleven high-power fields (400×) from three samples. Scale bar, 50 μm. Data were analyzed and visualized using R software.

Figure S3

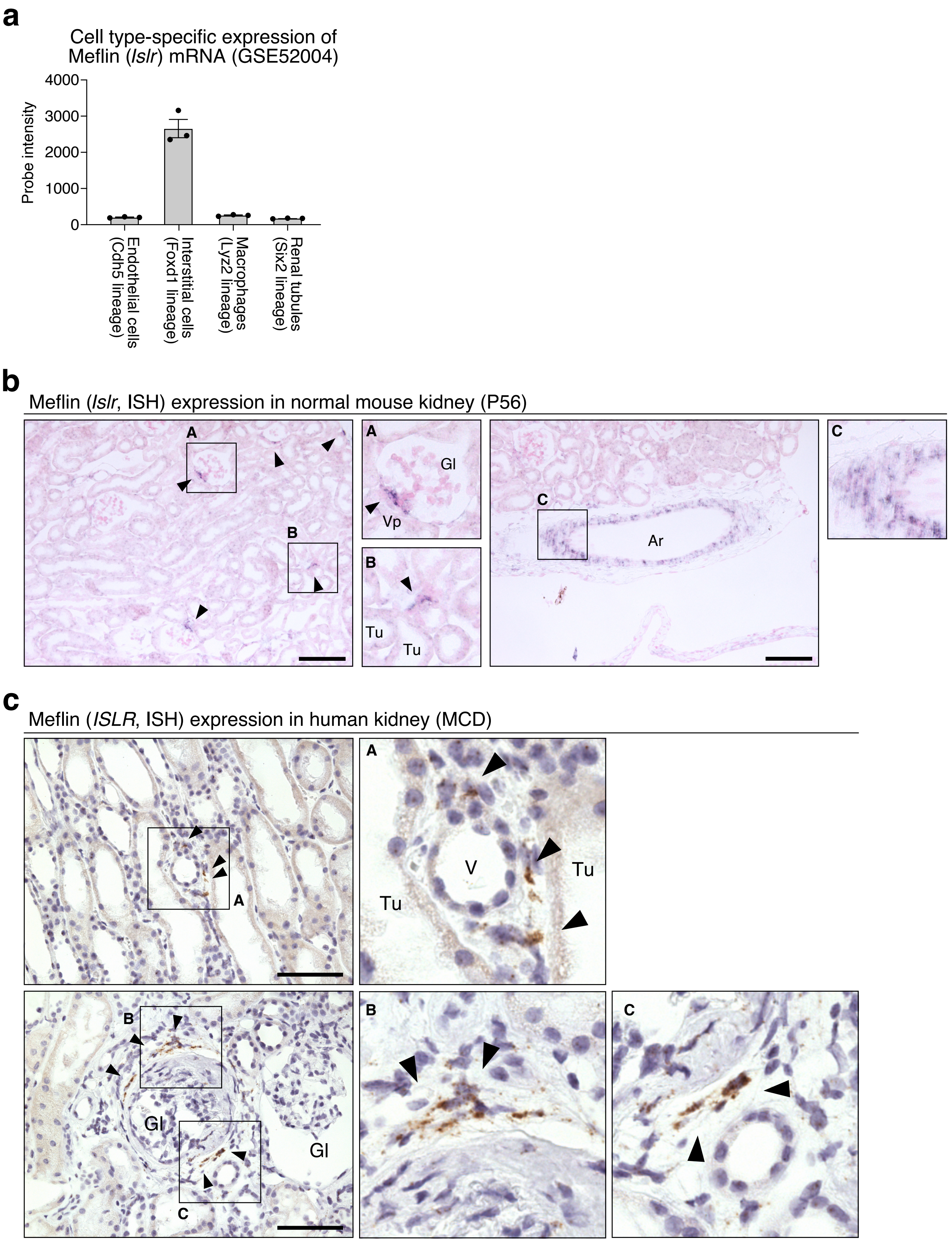
a
Cell type-specific expression of Meflin (Islr) mRNA (GSE52004)
Probe intensity
4000
3000
2000
1000
0
Endothelial cells (Cdh5 lineage)
Interstitial cells (Foxd1 lineage)
Macrophages (Lyz2 lineage)
Renal tubules (Six2 lineage)
b
Meflin (Islr, ISH) expression in normal mouse kidney (P56)
A
B
Gl
Vp
Tu
C
Ar
c
Meflin (ISLR, ISH) expression in human kidney (MCD)
V
Tu
Gl

Figure S3. Expression of Meflin (*Islr*) in the normal mouse and human kidney interstitial cells.

a. Cell type-specific expression of Meflin (*Islr*) in the normal mouse kidney (accession number: GSE52004). Foxd1-lineage interstitial cells showed the highest Meflin expression. Data were analyzed with GEO2R and are presented as the mean ± SEM. n = 3/group.

b, c. ISH for Meflin (*Islr*) in adult mouse (P56) (**b**) and human (**c**) kidneys. Human kidney tissue samples were obtained from a patient with minimal change disease (MCD). Boxed regions are magnified in adjacent panels. Arrowheads indicate cells that are positive for Meflin (*Islr*). Notably, Meflin^{+} PMCs were hardly detected in the glomerular vascular pole in the normal human kidney. The glomerulus shown in the lower panel is from a patient with MCD and mild adjacent sclerotic change. Boxed areas were magnified in adjacent panels. Scale bar, 100 μm. Gl, glomerulus; Tu, urinary tubules; Ar, artery; Vp, vascular pole; V, small vessels.

Figure S4

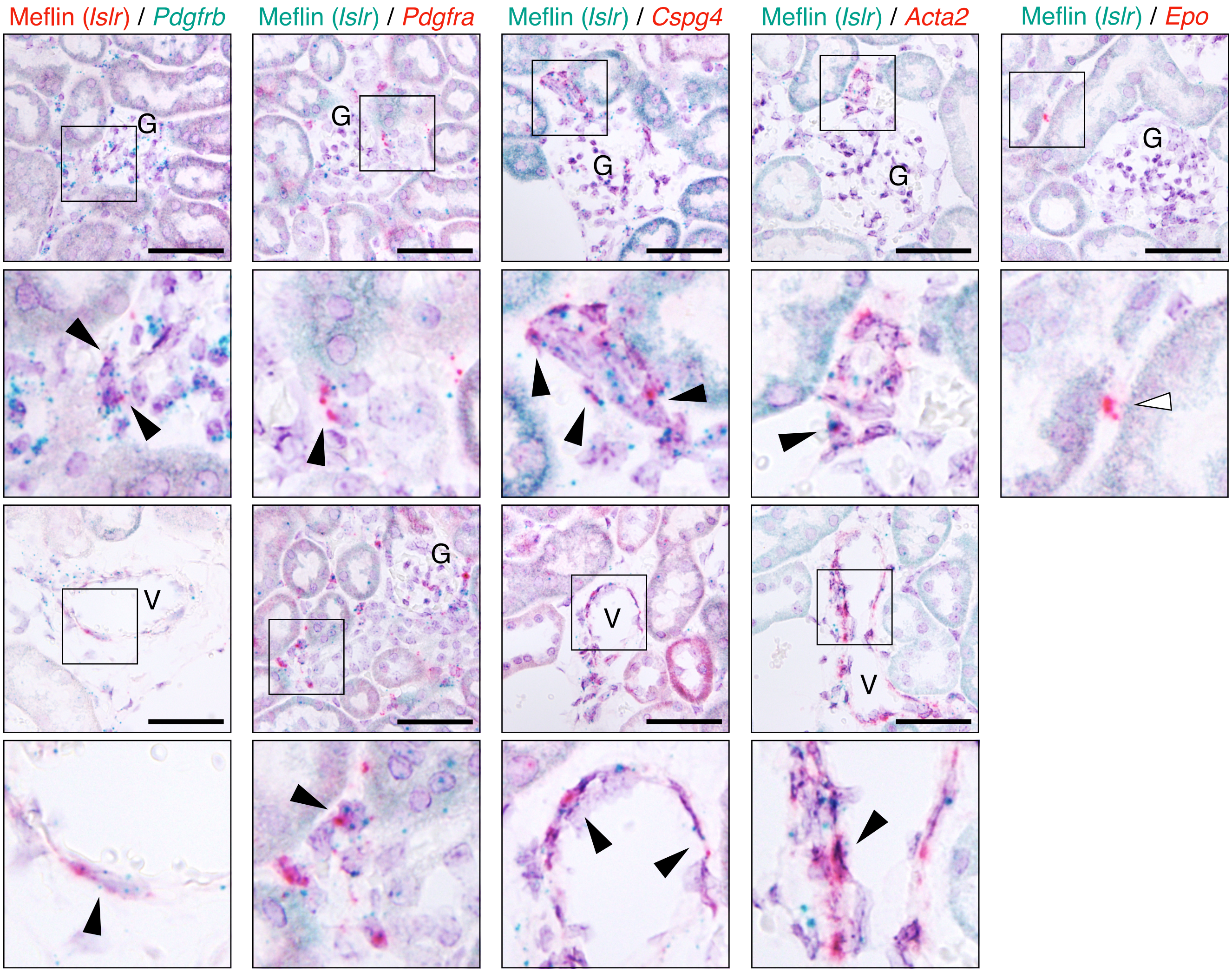
Meflin (Islr) / Pdgfrb
Meflin (Islr) / Pdgfra
Meflin (Islr) / Cspg4
Meflin (Islr) / Acta2
Meflin (Islr) / Epo
G
V

Figure S4. Co-expression of Meflin with classic pericyte markers in the mouse kidney.

Tissue sections of normal mouse kidney were double stained for Meflin and previously known pericyte markers using ISH. Meflin (*Islr*) mRNA was expressed in stromal cells positive for *Pdgfrb*, *Pdgfra*, *Cspg4*, and *Acta2* mRNA, suggesting that Meflin is a marker of pericytes or PMCs (black arrowheads). Notably, erythropoietin (encoded by the *Epo* gene), a marker of fibroblasts involved in renal fibrosis, was not co-expressed with Meflin (white arrowhead). Boxed areas were magnified in respective lower panels. Scale bar, 50 μm. G, glomerulus; V, small vessels.

Figure S5

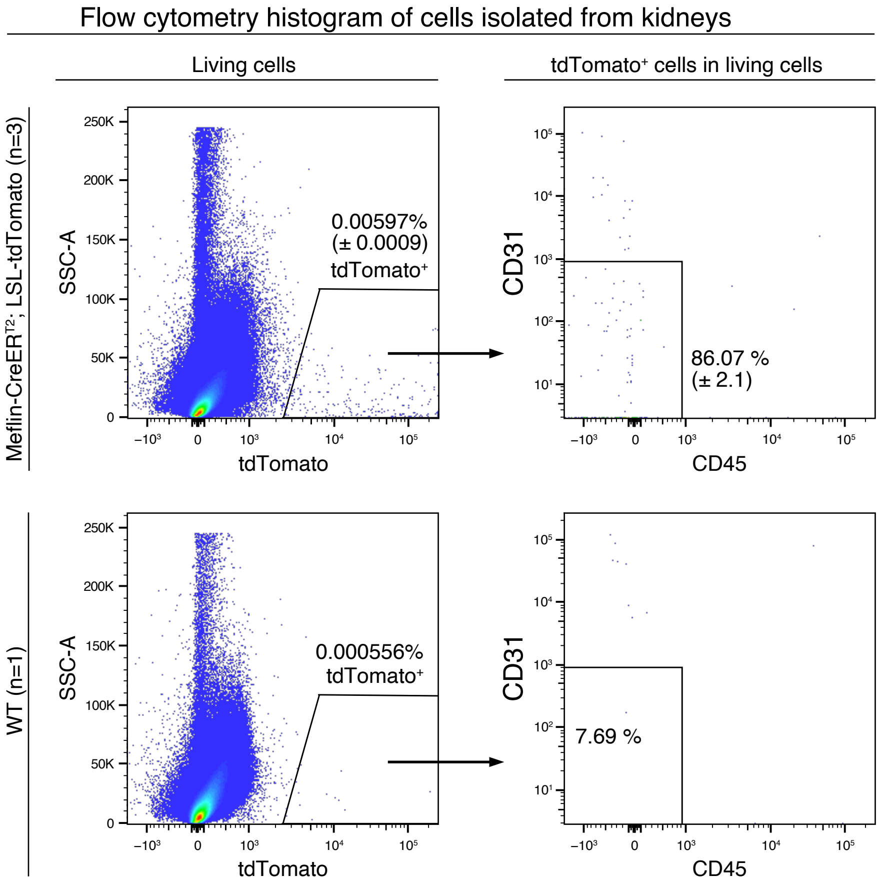

Flow cytometry histogram of cells isolated from kidneys
Living cells
tdTomato⁺ cells in living cells
Meflin-CreER^{T2}; LSL-tdTomato (n=3)
SSC-A
tdTomato
0.00597%
(± 0.0009)
tdTomato⁺
CD31
CD45
86.07 %
(± 2.1)
WT (n=1)
0.000556%
tdTomato⁺
7.69 %

Figure S5. Non-endothelial and non-hematopoietic cells express Meflin in the normal adult mouse kidneys.

All cells prepared from the kidneys of tamoxifen-administered Meflin-CreER^{T2}; LSL-tdTomato mice were sorted for the expression of tdTomato (upper left), followed by staining for the endothelial marker CD31 and the hematopoietic marker CD45. Note that most tdTomato^{+} cells (0.00597 ± 0.0009% of all cells) are negative for CD31 and CD45 (upper right). tdTomato^{+} cells were not detected in cells isolated from the kidneys of WT mice (lower panel), demonstrating specificity of the analysis.

Figure S6

Single-cell RNA seq of adult mouse healthy kidney (31,258 cells, GSE129798)

a

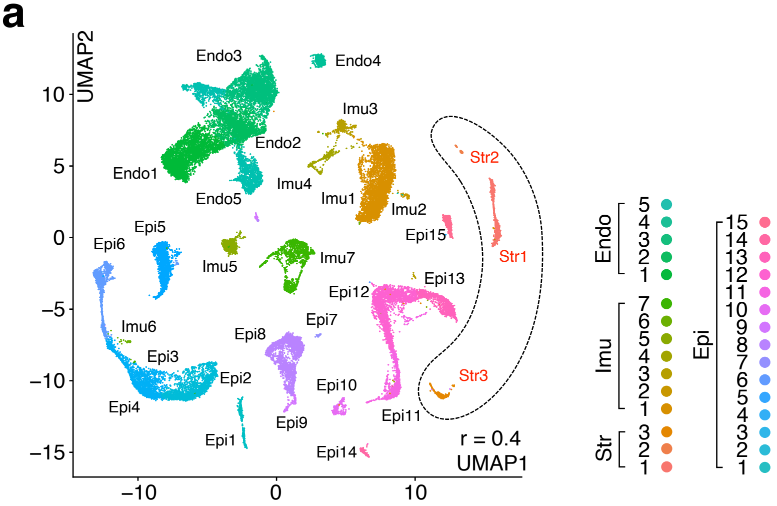

b

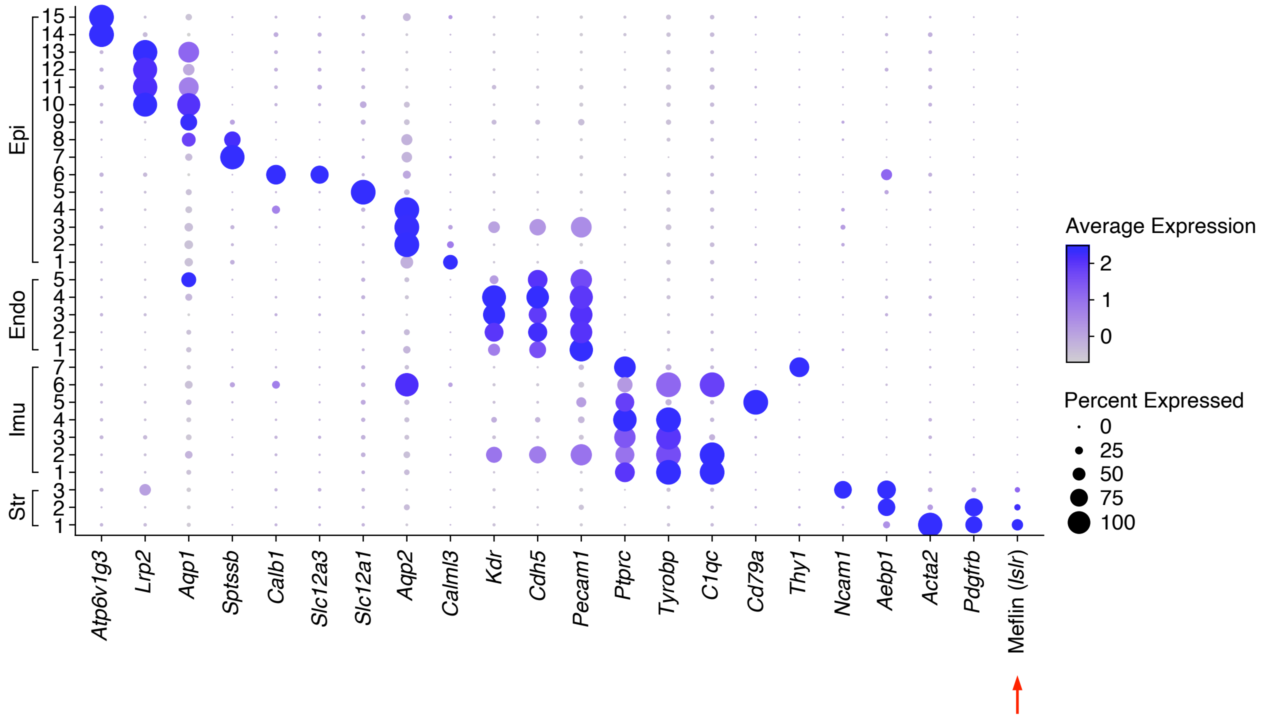

Figure S6. Expression of Meflin in stromal cell clusters within all cell clusters identified by single-cell RNA-seq analysis of the adult mouse healthy kidney.

a. Uniform manifold approximation and projection (UMAP) representation of all cell clusters identified by single-cell transcriptomic analysis of the normal adult kidneys (GSE129798). Three stromal cell clusters (Str1–3), seven immune cell clusters (Imu1–7), five endothelial cell clusters (Endo1–5), and fifteen epithelial cell clusters (Epi1–15) were identified. Stromal clusters are enclosed by a dashed line. r indicates clustering resolution.

b. Expression of marker genes representative of the indicated cell clusters according to single-cell transcriptomic analysis of the normal adult kidneys (GSE129798). The circle sizes refer to the percentage of cells expressing the respective genes in the indicated cell clusters, and the color intensities indicate expression levels of the respective genes. Note that Meflin (*Islr*) was specifically detected in the stromal clusters Str1, 2, and 3 but not other cell clusters (an arrow).

Data were analyzed and visualized using R software.

Figure S7

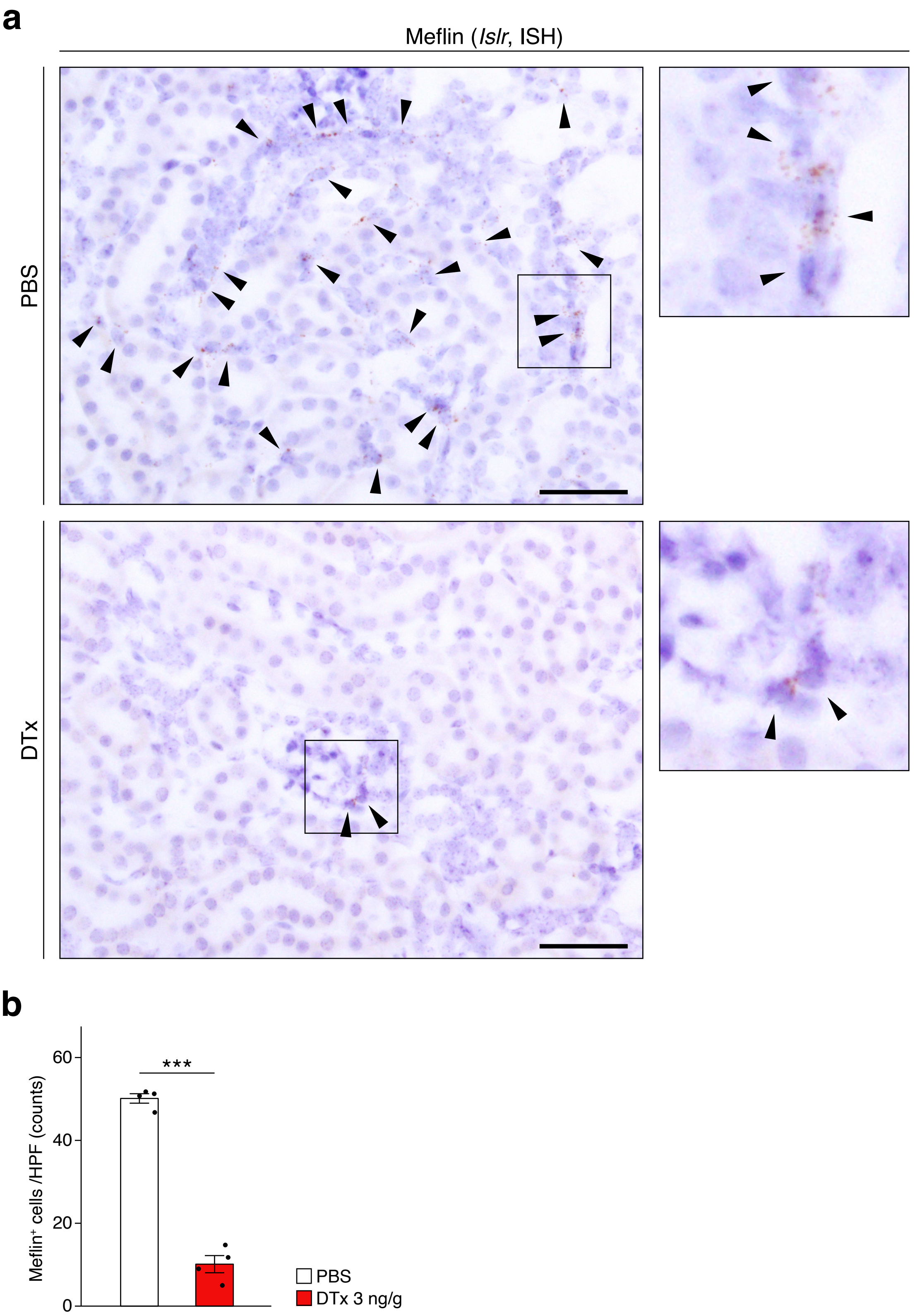
a
Meflin (Islr, ISH)
PBS
DTx
b
60
40
20
0
***
Meflin+ cells /HPF (counts)
PBS
DTx 3 ng/g

Figure S7. Genetic ablation of Meflin^{+} cells by DTx administration in Meflin-ZDC mice.

a. Meflin-ZDC mice (n = 4/group) treated with either PBS (upper) or diphtheria toxin (DTx, 3 ng/g; lower) were sacrificed three days after DTx administration, followed by preparation of kidney sections and ISH for Meflin (*Islr*). Meflin^{+} cells (arrowhead) were significantly decreased in DTx-administered mice compared to PBS-administered control mice. Boxed areas were magnified in adjacent panels. Scale bar, 50 μm.
b. The numbers of Meflin^{+} cells in the indicated groups were counted and quantified . Four HPF (400×) per each kidney were evaluated. Data are shown as the mean ± SEM. ***p < 0.001 (unpaired t-test with Welch's correction). Data were analyzed and visualized using R software.

Figure S8

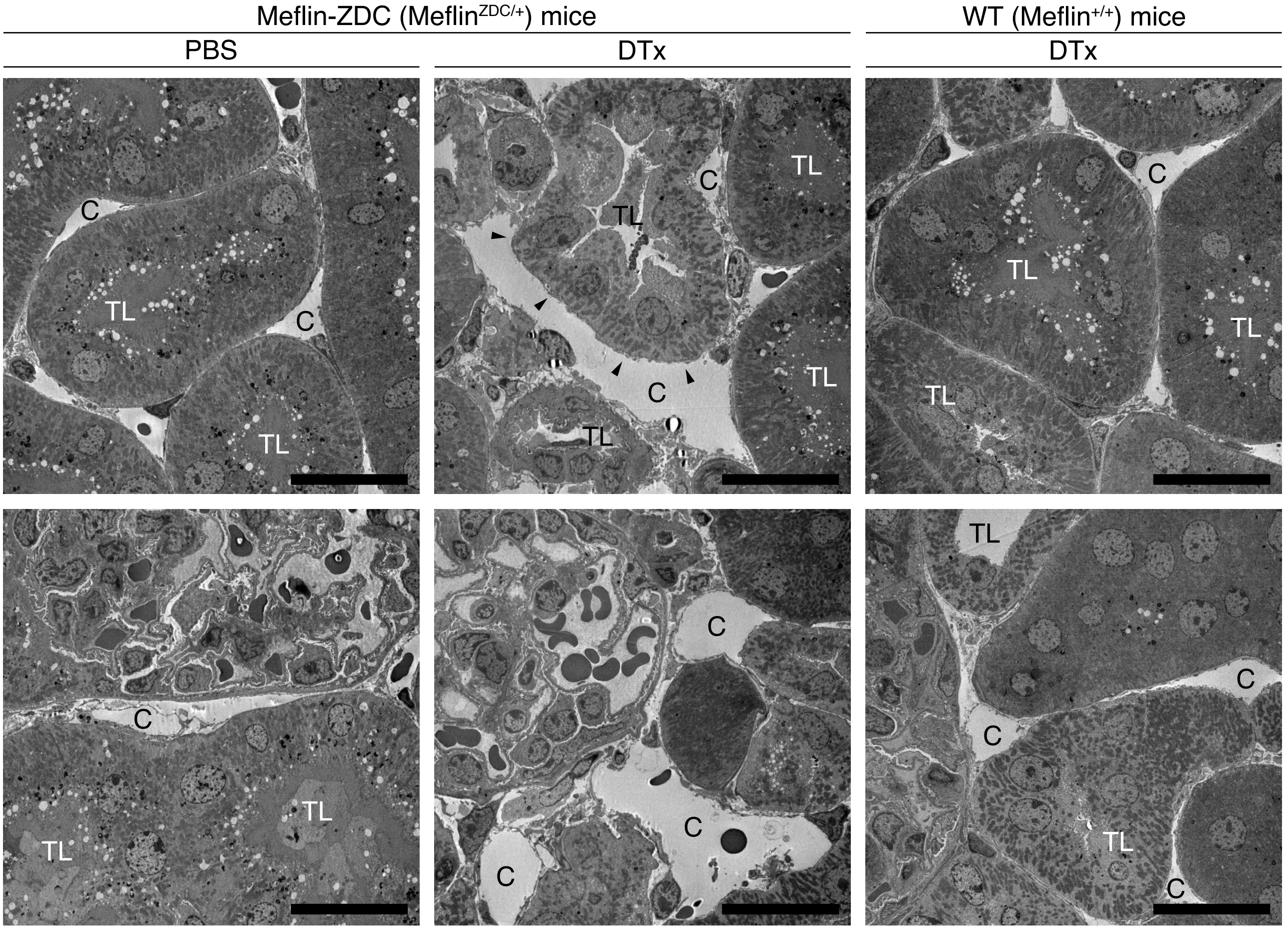

Meflin-ZDC (Meflin$^{ZDC/+}$) mice
WT (Meflin$^{+/+}$) mice
PBS
DTx
DTx
C
TL
C
TL
C
TL
C
TL
TL
C
TL
C
TL
TL
C
TL
TL
C
TL
C
C
C
C
TL
C
C
TL
C

Figure S8. Dilatation of capillaries and degeneration of tubular epithelial cells after ablation of Meflin^{+} PMCs.

Meflin-ZDC mice treated with either PBS (left) or diphtheria toxin (DTx, 3 ng/g; middle) and WT mice treated with DTx (right) were sacrificed three days after DTx administration, followed by transmission electron microscopy. Dilated capillaries and degenerated tubular epithelial cells (arrowheads) were observed in DTx-treated Meflin-ZDC mouse kidneys but not in other groups. Scale bar, 20 μm. C, capillary; TL, urinary tubular lumen.

Figure S9

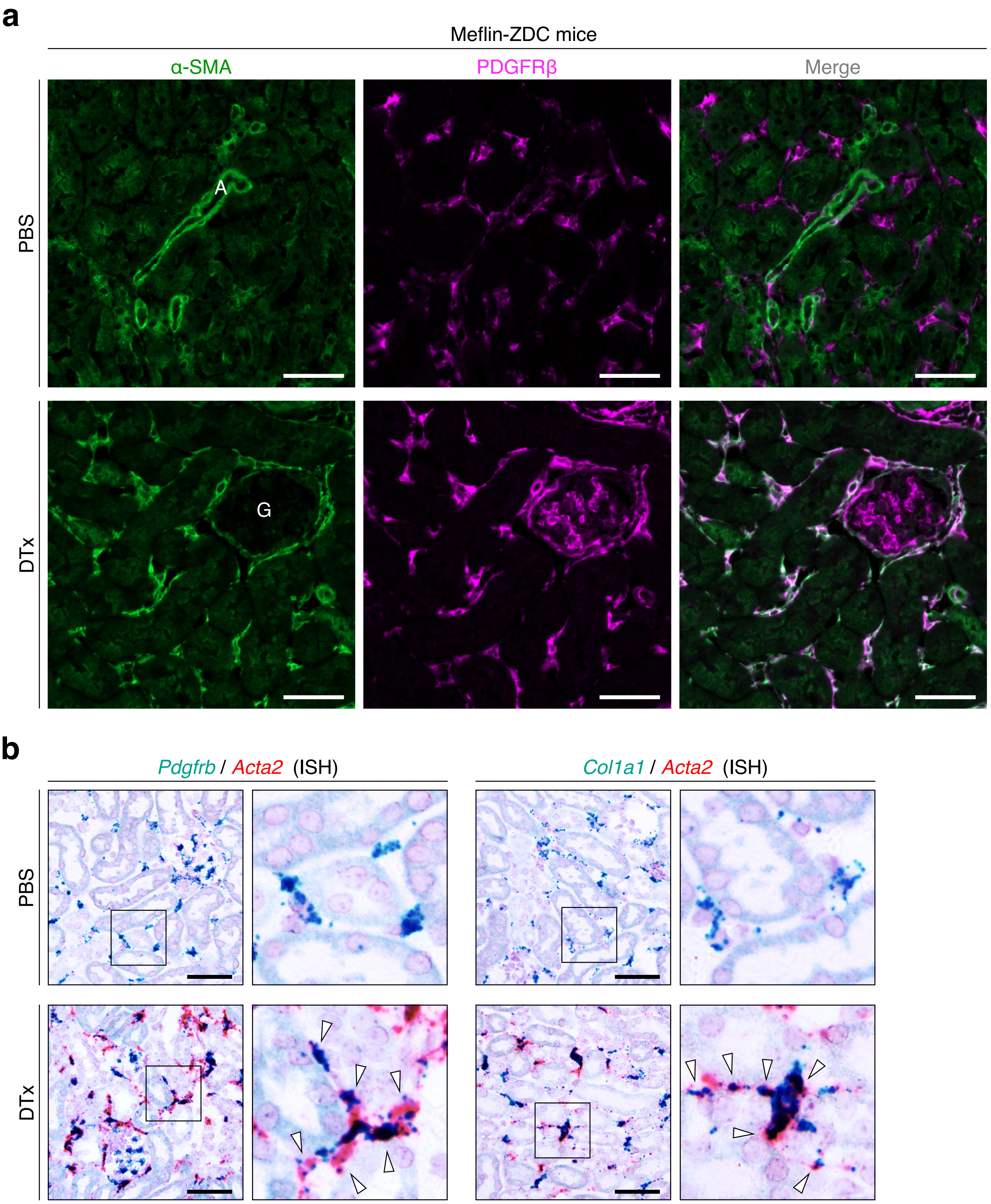
a
Meflin-ZDC mice
α-SMA
PDGFRβ
Merge
PBS
DTx
A
G
b
Pdgfrb / Acta2 (ISH)
Col1a1 / Acta2 (ISH)
PBS
DTx

Figure S9. PDGFRβ and collagen type I expression in α-SMA$^+$ cells that proliferate in the renal interstitial area of DTx-treated Meflin-ZDC mice.

a. Meflin-ZDC mice treated with either PBS (upper) or diphtheria toxin (DTx, 3 ng/g; lower) were sacrificed three days after DTx administration, followed by IF staining of kidney sections for α-SMA (green) and PDGFRβ (magenta). The data showed that most of α-SMA$^+$ cells proliferating in DTx-treated mice were positive for PDGFRβ. Scale bar, 50 µm. A, artery; G, glomerulus.

b. Kidney sections prepared from control (PBS) and DTx-administered Meflin-ZDC mice were double stained for *Acta2* and *Pdgfrb* (left) or *Acta2* and *Col1a1* (right) by ISH. *Acta2*$^+$ interstitial cells in DTx-treated mice were positive for both *Pdgfrb* and *Col1a1*. Arrowheads indicate the double positive cells. Boxed areas were magnified in adjacent panels. Scale bar, 50 µm.

Figure S10

a

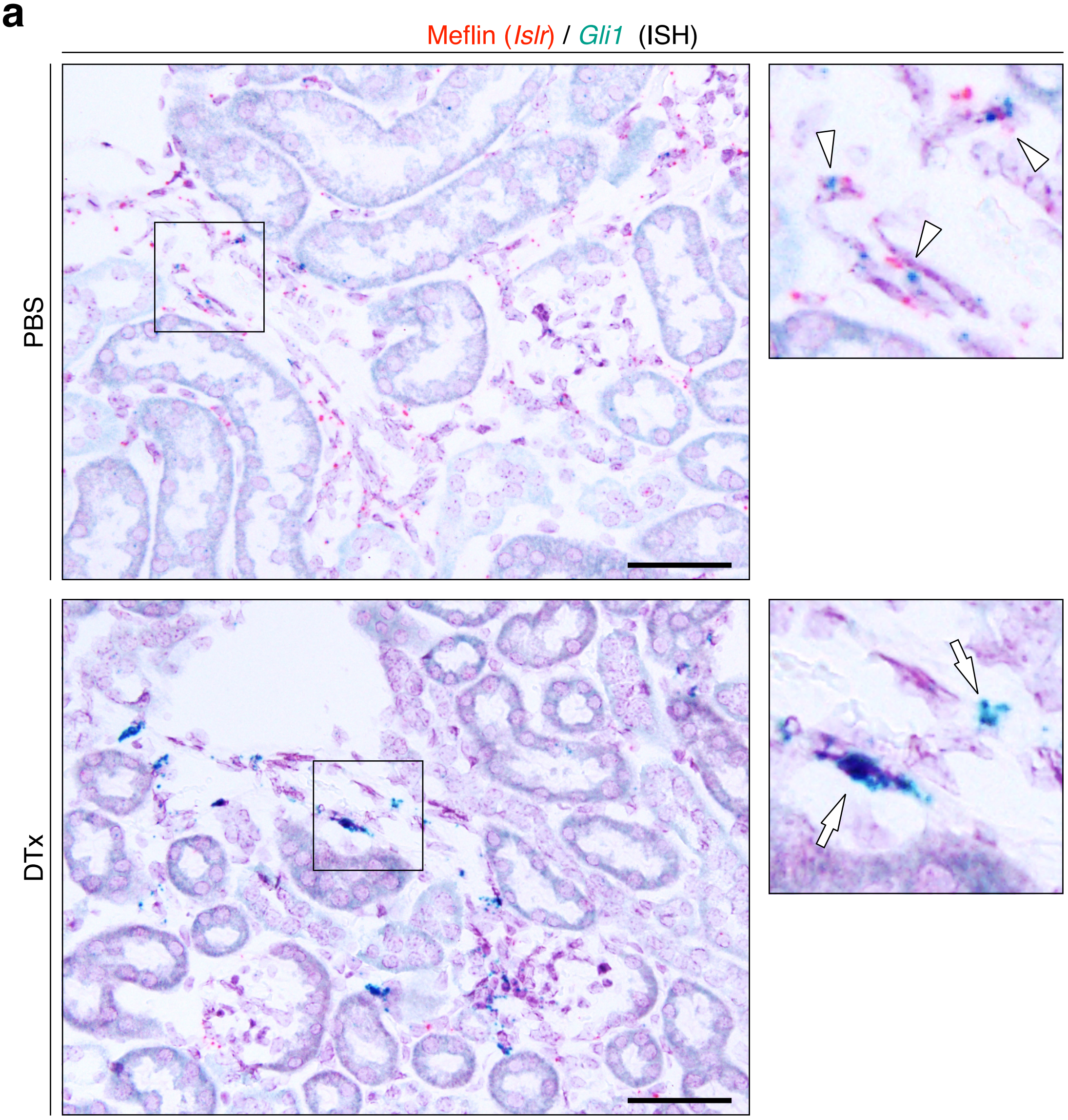

b

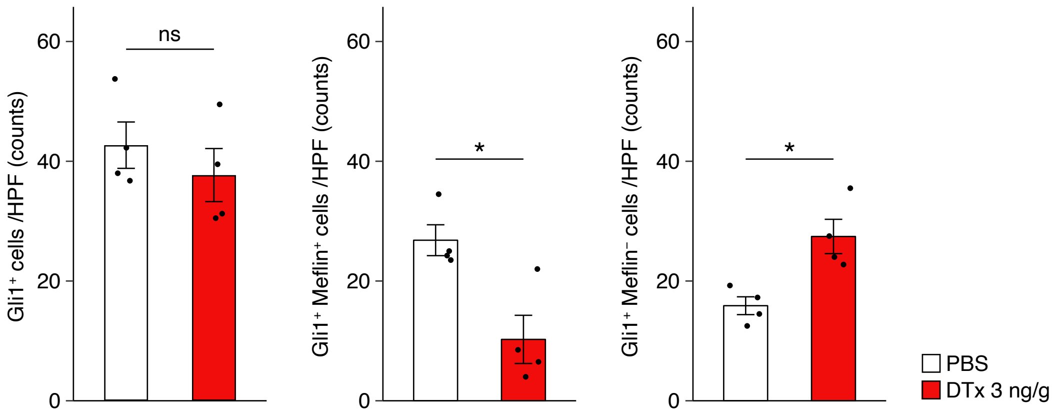

Figure S10. Reactive proliferation of *Gli1*$^{+}$Meflin^{-} cells after genetic ablation of Meflin^{+} cells in the kidney.

a. Meflin-ZDC mice (*n* = 4/group) treated with either PBS (upper) or diphtheria toxin (DTx, 3 ng/g; lower) were sacrificed three days after DTx administration, followed by double ISH for *Gli1* and Meflin (*Islr*) on kidney sections. *Gli1*$^{+}$Meflin^{+} cells were observed in PBS-administered control mice (arrowheads), whereas they were rare in DTx-administered mice. In DTx-administered mice, there was an increase in the numbers of *Gli1*$^{+}$Meflin^{-} cells (arrow), being accompanied by an increase in *Gli1* expression level. Boxed areas were magnified in adjacent panels. Scale bar, 50 μm.
b. The numbers of *Gli1*$^{+}$ (left), *Gli1*$^{+}$Meflin^{+} (middle), and *Gli1*$^{+}$Meflin^{-} (right) cells in the indicated groups were counted, followed by quantification. Four HPF (400×) per each kidney were evaluated. Data are shown as the mean ± SEM. *$p < 0.05$ (unpaired *t*-test with Welch's correction). Data were analyzed and visualized using R software.

Figure S11

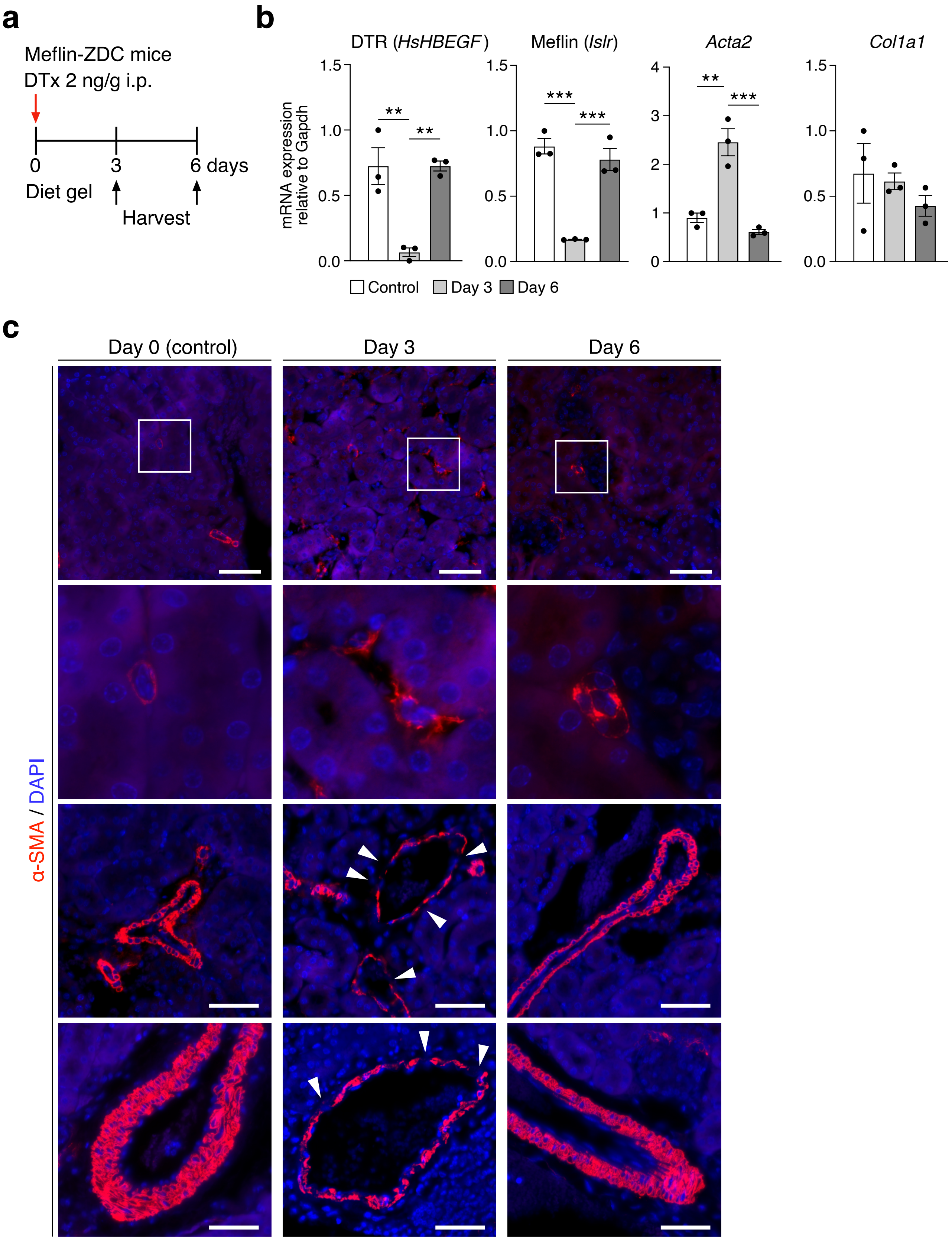

a
Meflin-ZDC mice
DTx 2 ng/g i.p.
0
3
6 days
Diet gel
Harvest
b
DTR (HsHBEGF)
Meflin (Islr)
Acta2
Col1a1
mRNA expression relative to Gapdh
1.5
1.0
0.5
0.0
4
3
2
1
0
**
***
Control
Day 3
Day 6
c
Day 0 (control)
Day 3
Day 6
α-SMA / DAPI

Figure S11. Time-dependent changes in Meflin and a-SMA expression and vasculature morphology in DTx-treated Meflin-ZDC mice.

a. Meflin-ZDC mice treated with DTx (2 ng/g, i.p.; n = 3/group) were sacrificed three and six days after DTx administration, followed by kidney harvesting for qPCR and immunofluorescence (IF) staining. These mice were fed with Diet gel to reduce gastrointestinal toxicity caused by ablating intestinal Meflin^{+} cells.
b. qPCR analysis of diphtheria toxin receptor (DTR, encoded by *HsHBEGF*) and Meflin (*Islr*), which were downregulated on day 3 but recovered on day 6. Note that α-SMA (*Acta2*) expression showed the opposite trend. Data are presented as mean ± SEM. $^{**}p < 0.01$, $^{***}p < 0.001$ (one-way ANOVA followed by Tukey's multiple comparisons test).
c. IF analysis for α-SMA showing the proliferation of α-SMA^{+} fibroblasts in the interstitial area (top two panels) on day 3, which was accompanied by vasculature dilatation (bottom two panels). Note that α-SMA^{+} vascular smooth muscle cells (VSMC) were discontinuously found in a mosaic pattern in the tunica media of the vessels on at day 3 (arrowheads). Boxed areas were magnified in lower panels. Scale bar, 50 μm.

Figure S12

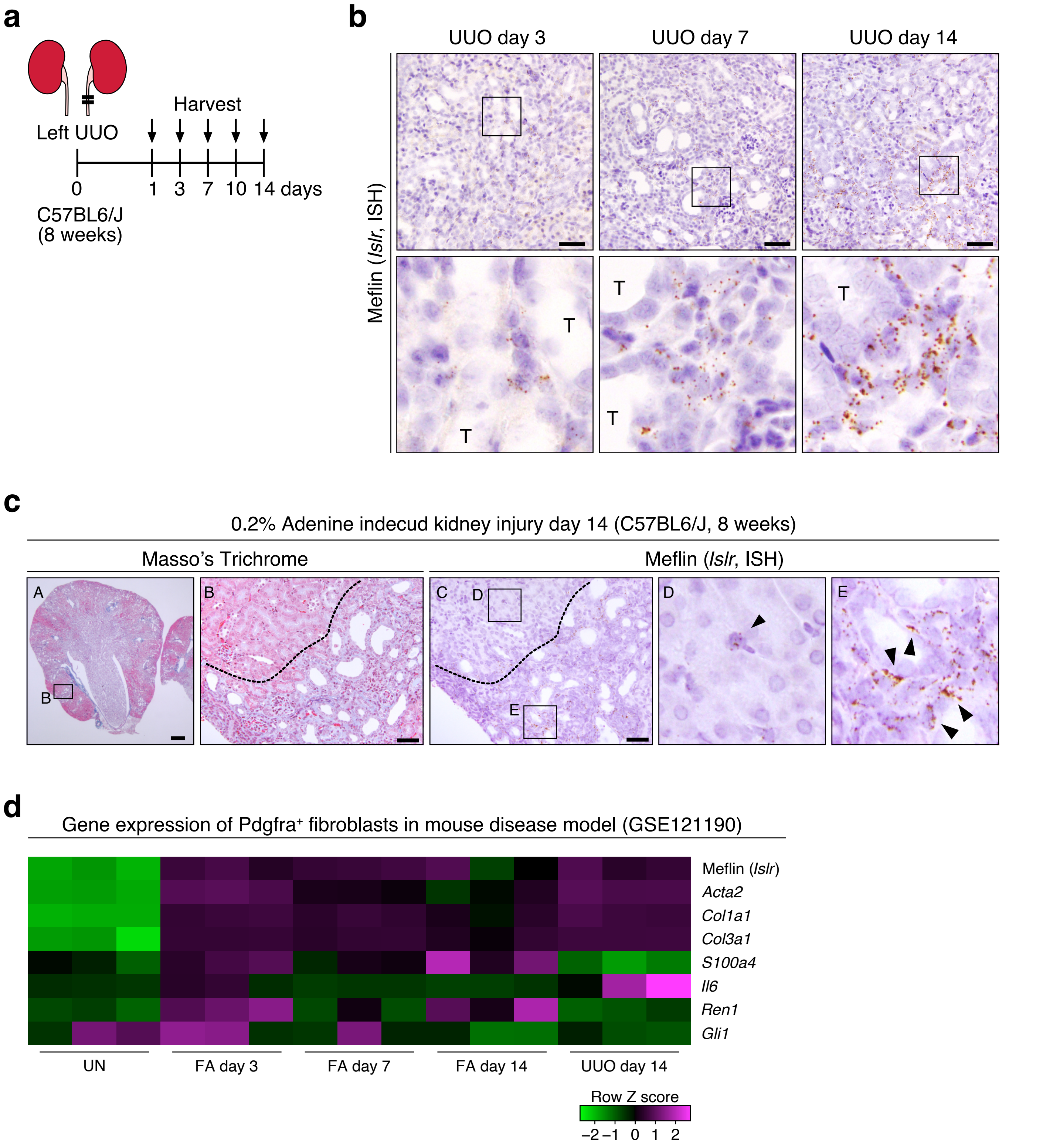
a
Harvest
Left UUO
0
1 3 7 10 14 days
C57BL6/J
(8 weeks)
b
UUO day 3
UUO day 7
UUO day 14
Meflin (Islr, ISH)
T
c
0.2% Adenine indecud kidney injury day 14 (C57BL6/J, 8 weeks)
Masso's Trichrome
Meflin (Islr, ISH)
A
B
C
D
E
d
Gene expression of Pdgfra+ fibroblasts in mouse disease model (GSE121190)
Meflin (Islr)
Acta2
Col1a1
Col3a1
S100a4
Il6
Ren1
Gli1
UN
FA day 3
FA day 7
FA day 14
UUO day 14
Row Z score
−2 −1 0 1 2

Figure S12. Proliferation of Meflin⁺ PMCs in renal fibrosis and injury mouse models.

a. Adult (P56) WT C57BL6/J mice underwent UUO surgery and then sacrificed on the indicated days after surgery.
b. ISH showing the proliferation of Meflin$^+$ PMCs in the interstitial area of UUO kidneys. Boxed regions are magnified in adjacent panels. Scale bar, 50 μm. T, renal tubules.
c. Adult (P56) WT C57BL6/J mice underwent adenine-induced kidney injury. Masson's trichrome staining showing a patchy fibrotic area (**A, B**). ISH of Meflin$^+$ PMCs (arrowheads), which significantly proliferated in the fibrotic area (**E**) but were sparsely distributed in the intact area (**D**). Boxed regions are magnified in adjacent panels. Scale bars, 400 (**A**) and 50 (**B**, **C**) μm.
d. Gene expression profiles of proliferative PDGFRα^+ fibroblasts (GSE121190) in the folic acid (FA)-induced kidney injury and UUO renal fibrosis models. Meflin (*Islr*), *Acta2*, *Col1a1*, and *Col3a1* expression was upregulated in these models, suggesting their involvement in the etiology of kidney diseases. Data were analyzed with GEO2R and visualized as Z scores using R software.

Figure S13

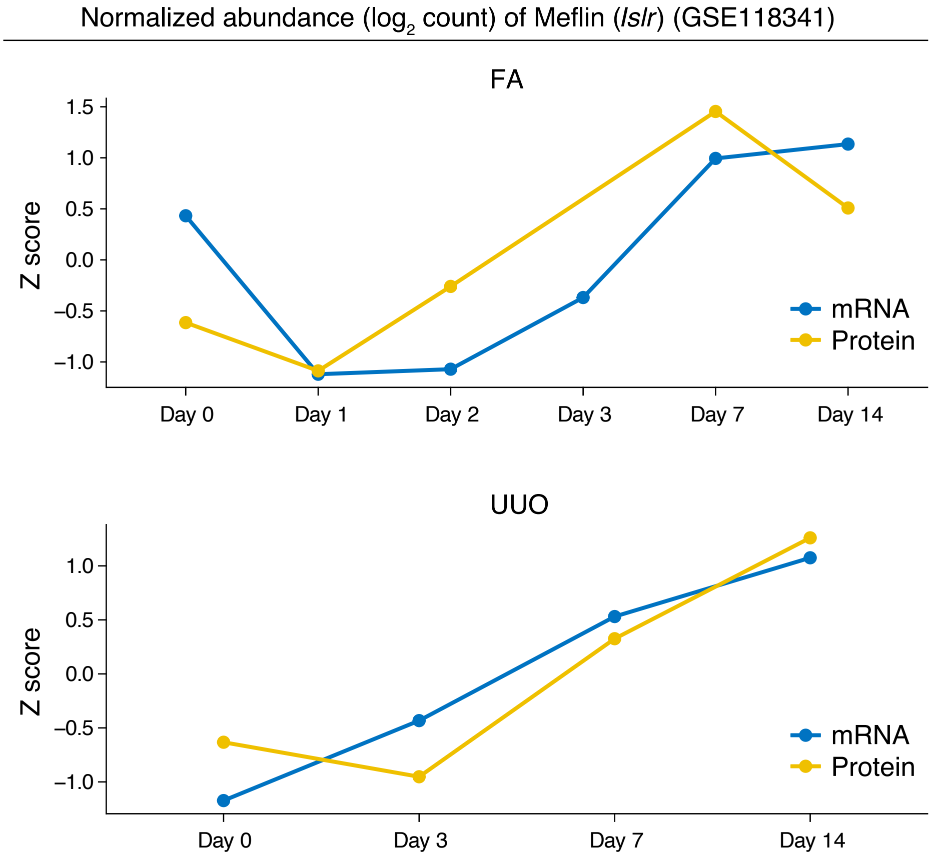

Normalized abundance (log_2 count) of Meflin (*Islr*) (GSE118341)
FA
Z score
1.5
1.0
0.5
0.0
−0.5
−1.0
Day 0
Day 1
Day 2
Day 3
Day 7
Day 14
mRNA
Protein
UUO
Z score
1.0
0.5
0.0
−0.5
−1.0
Day 0
Day 3
Day 7
Day 14
mRNA
Protein

Figure S13. Induced expression of Meflin at the mRNA and protein levels in renal fibrosis and injury mouse models.

Multi-omics dataset of diseased mouse kidneys (GSE118341) showed significant, time-dependent increases in Meflin mRNA (blue) and protein (yellow) levels in FA-induced kidney injury (upper panel) and UUO-induced kidney fibrosis (lower panel) models. Data were analyzed with GEO2R and visualized as Z scores using R software.

Figure S14

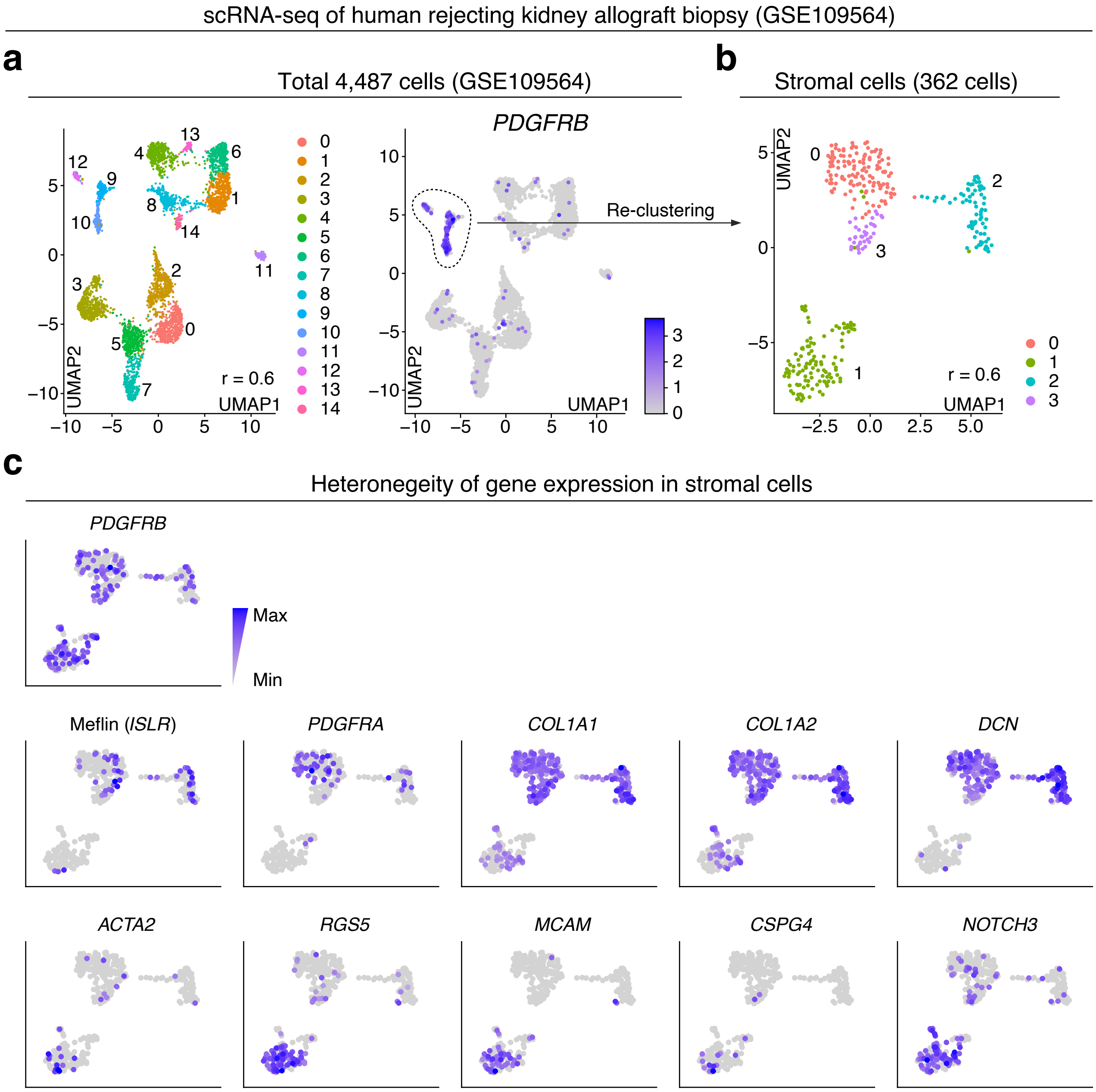
scRNA-seq of human rejecting kidney allograft biopsy (GSE109564)
a
Total 4,487 cells (GSE109564)
PDGFRB
Re-clustering
UMAP1
UMAP2
r = 0.6
b
Stromal cells (362 cells)
c
Heteronegeity of gene expression in stromal cells
PDGFRB
Max
Min
Meflin (ISLR)
PDGFRA
COL1A1
COL1A2
DCN
ACTA2
RGS5
MCAM
CSPG4
NOTCH3

Figure S14. Heterogeneous expression of Meflin in proliferative fibroblasts in human kidneys.

Inverse correlation between Meflin and α-SMA expression in fibroblasts of human diseased kidneys. UMAP plot with the distinct 15 cell populations identified by single-cell transcriptomic analysis of 4,487 cells isolated from human allograft kidney tissues undergoing clinical rejection (GSE109564) (**a**). UMAP plot of *PDGFRB* expression in the stromal cell population (the area enclosed by a dashed line; right panel), which was further expanded in (**b**) and re-clustered, which identified four (0–3) subsets of stromal cells. UMAP plots in (**c**) show gene expression in the four stromal subsets. Note that Meflin is preferentially expressed in stromal cells that are different from those highly expressing α-SMA (*ACTA2*) and conventional pericyte markers including *RGS5*, *MCAM*, and *CSPG4*. r indicates clustering resolution. Data were analyzed and visualized using R software.

Figure S15

a

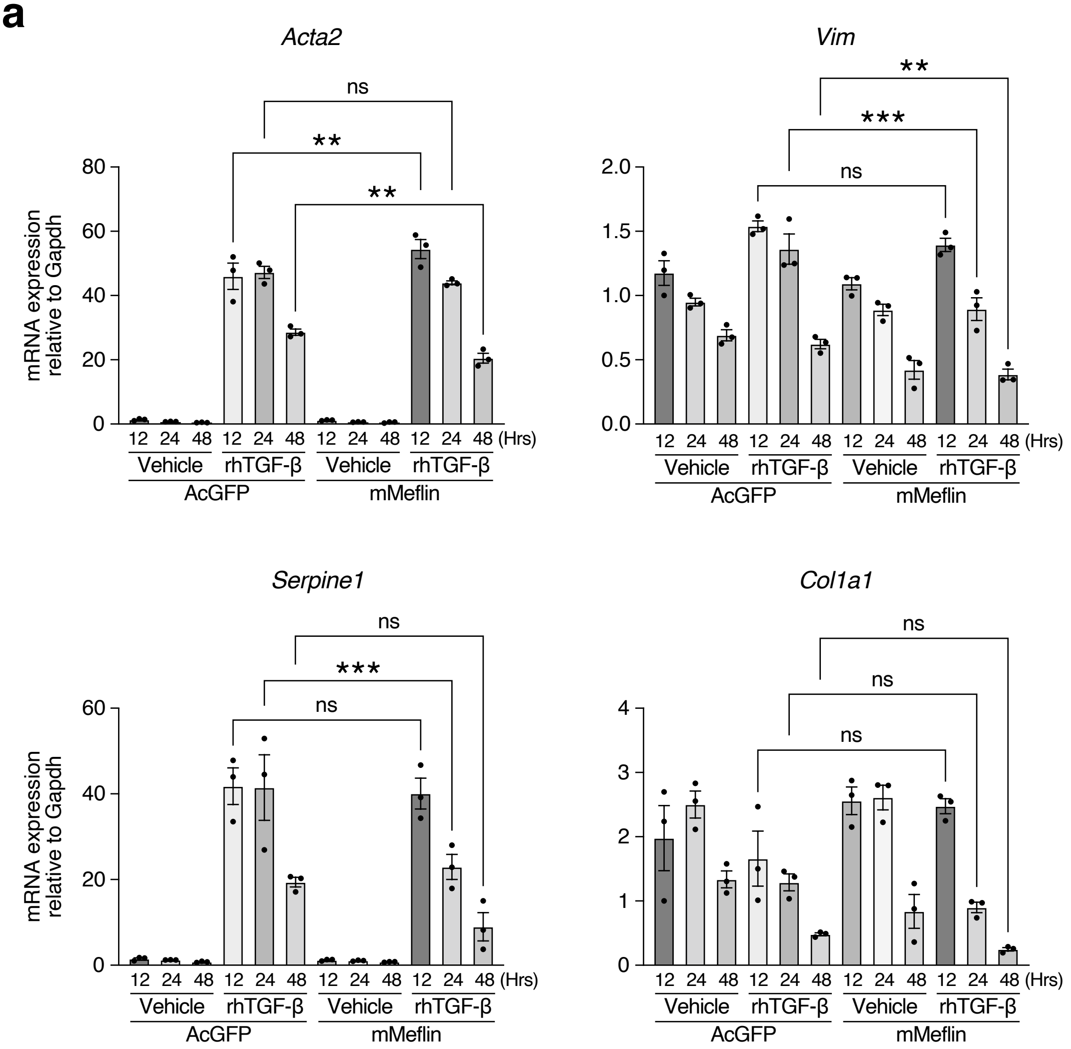

b

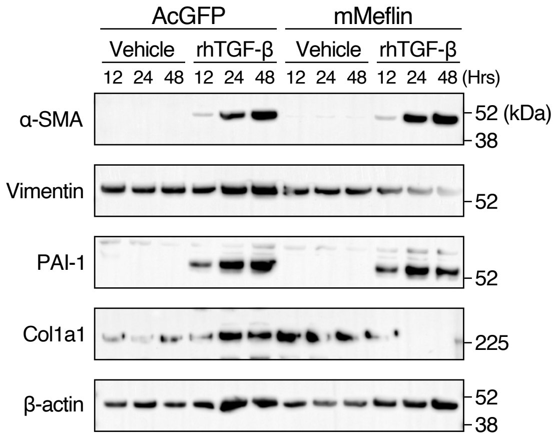

Figure S15. Effects of Meflin overexpression on TGF-β-mediated changes in gene and protein expressions in kidney fibroblasts.

NRK-49F cells transduced with AcGFP (control) or mouse Meflin (mMeflin) were stimulated with recombinant human TGF-β (2 ng/mL) for 12, 24, and 48 h, followed by qPCR for the indicated genes (n = 3/group) (**a**) and Western blotting for the indicated proteins (**b**).

*$p < 0.05$, **$p < 0.01$, ***$p < 0.001$ (one-way ANOVA followed by Sidak's multiple comparisons test in **a**).

Table S1. Characteristics of 41 patients who underwent renal biopsy

**Characteristics**	MCNS, *n* = 10	DMN, *n* = 11	IgAN, *n* = 20
**Age**, yr.	38 (29, 51)	70 (62, 72)	48 (39, 52)
**Sex**			
Male	6 (60%)	8 (73%)	10 (50%)
Female	4 (40%)	3 (27%)	10 (50%)
**BUN**, mg/dL	17 (14, 18)	17 (14, 23)	17 (14, 24)
**Cre**, mg/dL	0.85 (0.70, 1.01)	1.23 (0.95, 1.80)	1.00 (0.80, 1.44)
**Proteinuria**, g/gCr	10.8 (7.0, 12.2)	1.6 (1.1, 2.1)	1.6 (0.6, 2.7)

Values represent median (interquartile range); n (%)
BUN, blood urea nitrogen; Cre, creatinine; DKD, diabetic kidney disease; IgAN, IgA nephropathy; MCNS, minimal change nephrotic syndrome

Table S2. Characteristics of 99 patients with IgAN

Characteristics	*n*	Overall<br>*n* = 99	Meflin low<br>*n* = 80	Meflin high<br>*n* = 19	*p*-value[A]
**Sex**	99				0.7
Male		43 (43%)	34 (42%)	9 (47%)	
Female		56 (57%)	46 (57%)	10 (53%)	
**Age**, yr.	99	33 (25, 46)	33 (25, 45)	40 (26, 58)	0.2
**BMI**	99	21.6 (19.7, 24.1)	21.5 (19.6, 24.1)	22.1 (19.8, 23.6)	0.7
**HT**	99	32 (32%)	25 (31%)	7 (37%)	0.6
**DM**	98	4 (4.1%)	3 (3.8%)	1 (5.3%)	>0.9
**Urine occult blood**	99				0.041
0		16 (16%)	11 (14%)	5 (26%)	
1+		9 (9.1%)	8 (10%)	1 (5.3%)	
2+		27 (27%)	26 (32%)	1 (5.3%)	
3+		47 (47%)	35 (44%)	12 (63%)	
**Proteinuria**, g/day	99	0.98 (0.58, 1.69)	0.96 (0.53, 1.60)	1.52 (0.83, 2.27)	0.038
**Cre**, mg/dL	99	0.80 (0.67, 1.01)	0.80 (0.64, 1.00)	0.90 (0.77, 1.17)	0.02
**eGFR**, ml/min/1.73m^2	99	74 (57, 92)	77 (61, 93)	61 (45, 76)	0.009
**Steroid treatment**[B]	98	71 (72%)	58 (72%)	13 (72%)	>0.9
**RASi**	99	77 (78%)	60 (75%)	17 (89%)	0.2
**Observation period**, yr.	99	6.2 (4.1, 9.6)	6.5 (4.2, 9.6)	5.7 (3.2, 8.2)	0.2
**30% decline in eGFR**	99	16 (16%)	9 (11%)	7 (37%)	0.013

Value represent Median (Interquartile range); n (%)

BMI, body mass index; HT, hypertension; DM, diabetes mellitus; Cre, creatinine; eGFR, estimated glomerular filtration rate; RASi, renin-angiotensin system inhibitor (angiotensin-converting enzyme inhibitors or angiotensin receptor blockers)

[A] Pearson's Chi-squared test; Wilcoxon rank sum test; Fisher's exact test (between Meflin low vs high)

[B] Steroid treatment by Pozzi’s regimen
