# Supplementary material for: A novel renal perivascular mesenchymal cell subset gives rise to fibroblasts distinct from classic myofibroblasts
Source: Sci Rep. 2022 Mar 30;12:5389. doi: 10.1038/s41598-022-09331-5 (PMC8967907; doi:10.1038/s41598-022-09331-5)
Supplement: Supplementary file 2 — Supplementary Information 2. [file 41598_2022_9331_MOESM2_ESM.pdf]

Full unedited gel for Western blot data

Figure S1

Upper lane (Meflin)

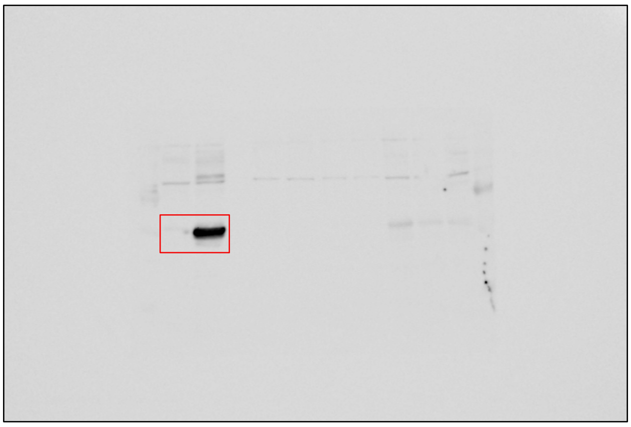

Lower lane (β-actin)

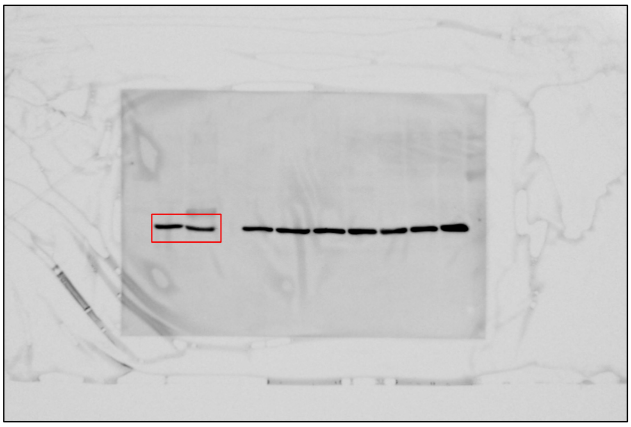

Meflin (Raw data with lower exposure)

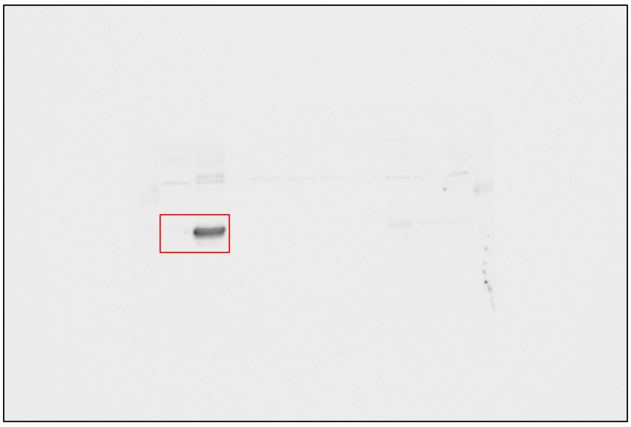

β-actin (Raw data with lower exposure)

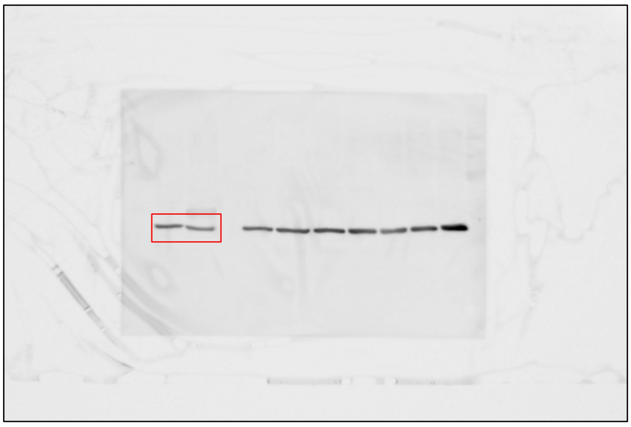

Red squares indicate cropped area.

Figure S15b

α-SMA

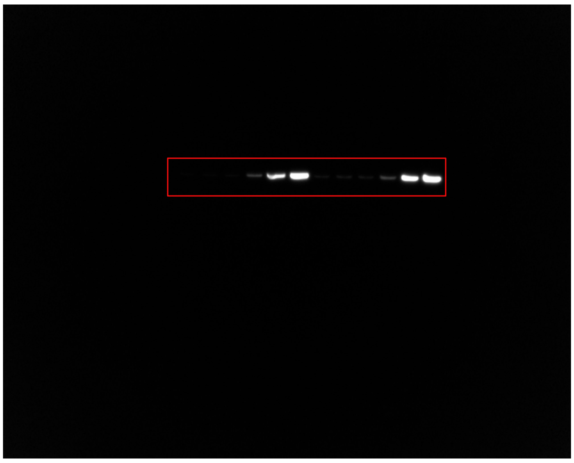

Vimentin

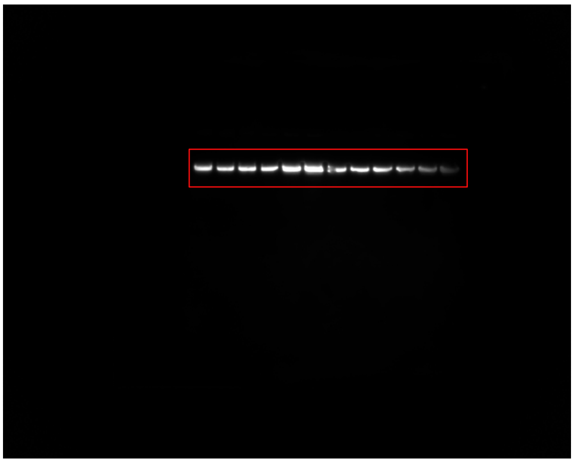

PAI-1

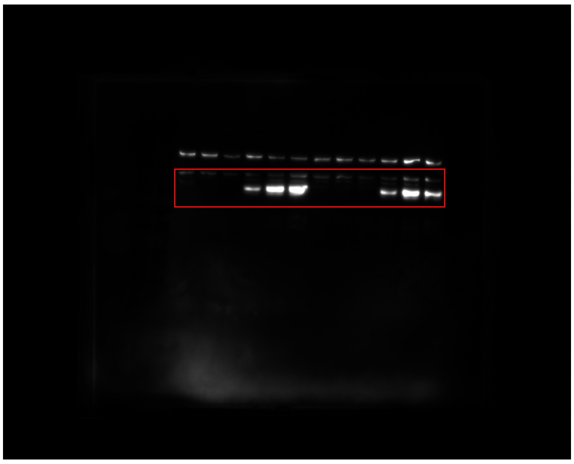

Col1a1

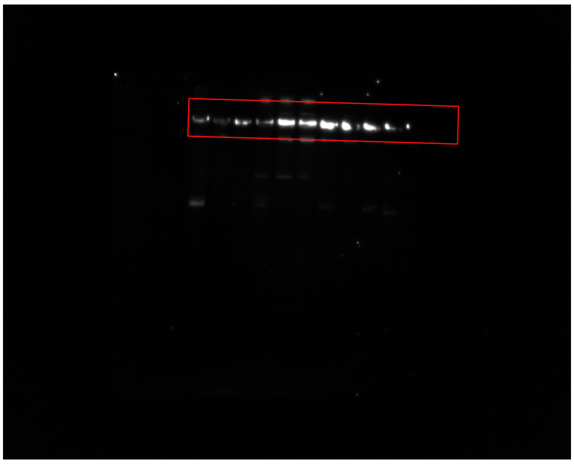

β-actin

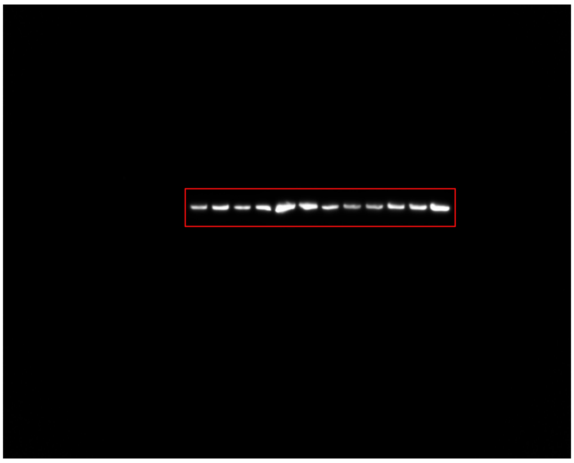

Red squares indicate cropped area.

Raw data with lower exposure

α-SMA

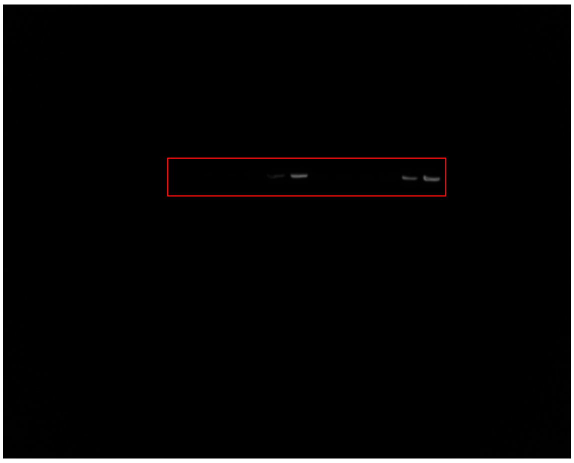

Vimentin

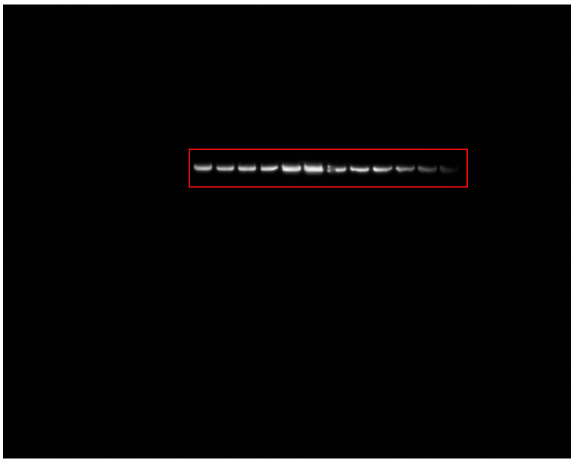

PAI-1

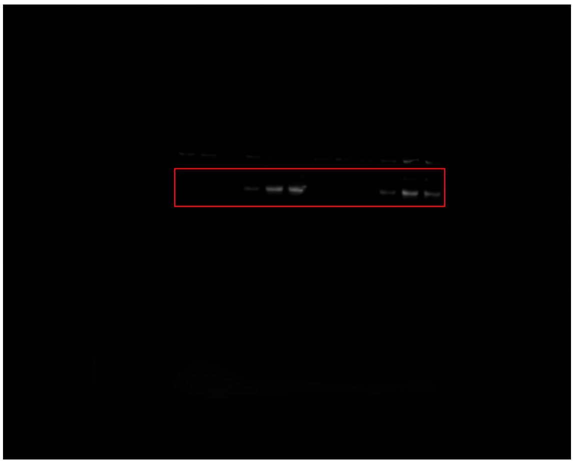

Col1a1

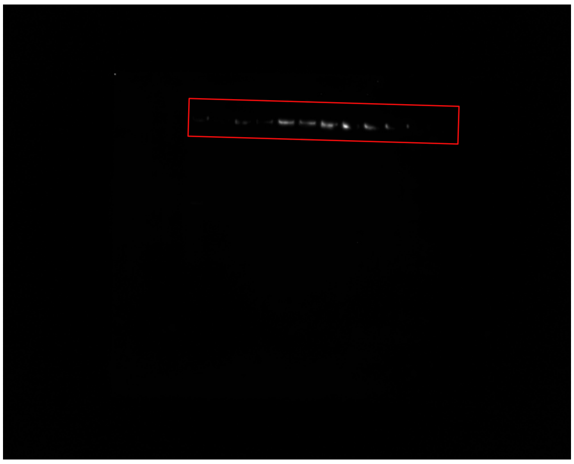

β-actin

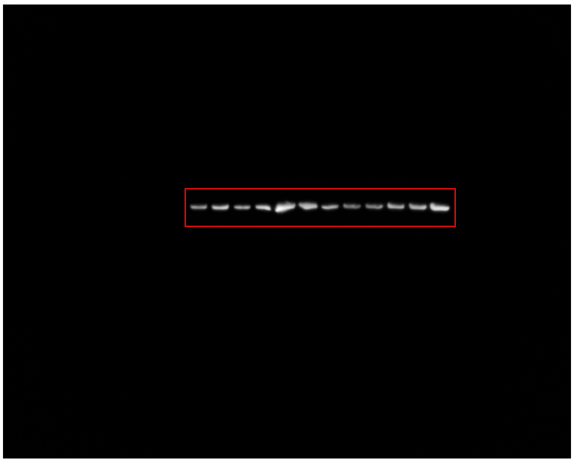

Red squares indicate cropped area.
